# Long-Range Allosteric Modulation of DNA Duplex Dynamics Induced by Pyrrole-Imidazole Polyamide Binding

**DOI:** 10.1021/acs.jpclett.5c01542

**Published:** 2025-07-28

**Authors:** Sophie E. T. Kendall-Price, Ryan J. O. Nichol, Andrea Taladriz-Sender, Ryan Phelps, Partha Malakar, Gregory M. Greetham, Glenn A. Burley, Neil T. Hunt

**Affiliations:** † Department of Chemistry and York Biomedical Research Institute, 8748 University of York, Heslington, York YO10 5DD, U.K.; ‡ Department of Pure and Applied Chemistry, University of Strathclyde, Glasgow G1 1BX, U.K.; § STFC Central Laser Facility, Research Complex at Harwell, Harwell Science and Innovation Campus, Didcot OX11 0QX, U.K.

## Abstract

The allosteric modulation of the structural dynamics of double-stranded DNA (dsDNA) duplexes as a function of distance from the site of a minor groove binding ligand is reported. Time-resolved temperature-jump infrared spectroscopy is used to interrogate the impact of binding a pyrrole-imidazole polyamide to dsDNA sequences 8–14 base-pairs in length. Our results demonstrate that the binding of the hairpin polyamide to its target site (5′-WWGTACW-3′; W = A/T) causes a marked suppression of structural dynamics, such as end fraying, with suppression observed in both the 3′ and 5′ directions. Quantitative analysis of end fraying suppression reveals a propagation length for dynamic modulation of 30 base-pairs. Identifying the structural impact of minor groove binding to dsDNA sequences furthers our understanding of the influence of dsDNA recognition and informs the design of next-generation synthetic transcription factors.

## Graphical abstract

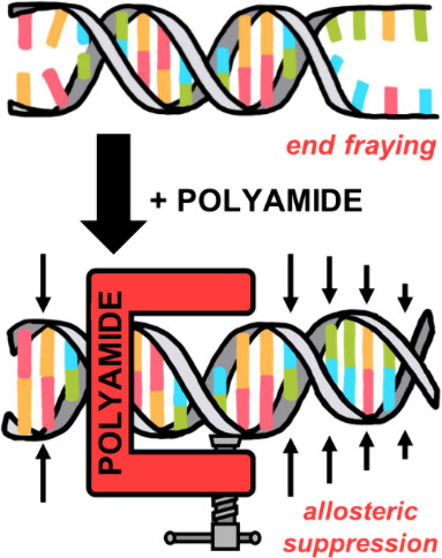

Sequence selective recognition of double-stranded DNA (dsDNA) is essential for transcriptional initiation. In many cases, recruitment of more than one DNA binding protein to a dsDNA sequence is required and evidence of cooperative protein binding at remote sites, mediated by allosteric interactions, underpins many aspects of transcriptional regulatory processes.^1–3^ While binding to dsDNA can result in structural changes leading to local allosteric modulation effects, longer range examples of allostery can occur without a corresponding conformational change in duplex structure. Although the mechanism for this remains unclear, it has been suggested that ligand-induced changes to the dynamics of dsDNA may underpin such allosteric communication mechanisms.^4–7^ This topic has been discussed in detail elsewhere.^8–10^

To shed light on this, we report the impact upon dsDNA duplex structural dynamics arising from the recognition of a target sequence by a pyrrole–imidazole polyamide (PA). PAs modulate transcription both *in vitro* and *in vivo* by virtue of their programmable, sequence-selective, binding.^11–16^ When in complex with dsDNA, PAs can adopt a hairpin conformation in which pyrrole–pyrrole (Py-Py) pairings recognize A/T sequences (W), whereas pyrrole-imidazole (Py-Im) units recognize C-G sequences with Im-Py units recognizing G-C (Figure 1(a,b)). Upon minor groove binding, PAs induce local allosteric compression of the major groove on the opposite face of the binding site, while concomitantly widening the minor groove. Here, we investigate whether PA binding also gives rise to additional allosteric disruption at remote locations on dsDNA.

**Figure 1:**
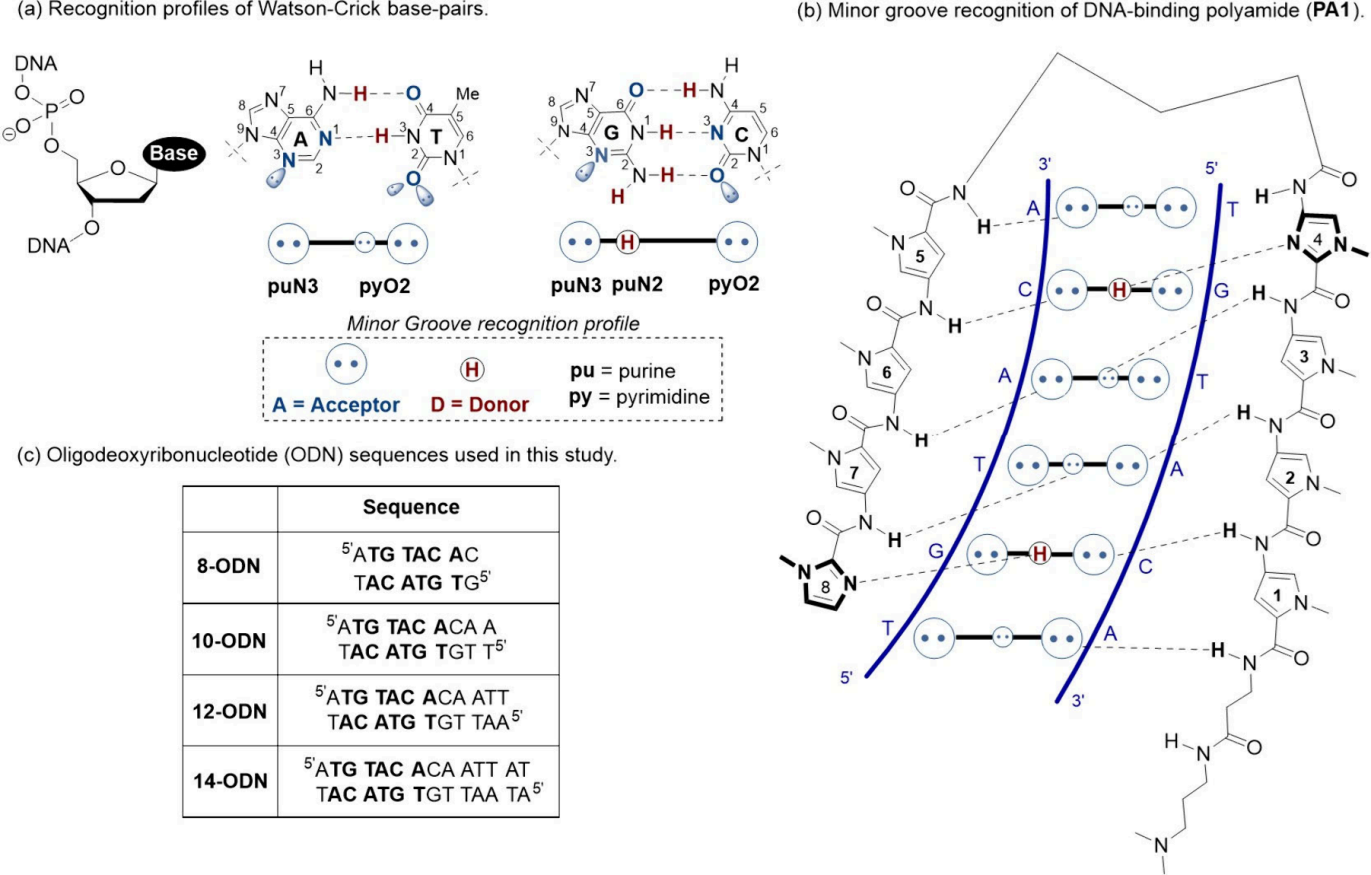
(a) Schematic diagram showing nomenclature used to describe the interaction of dsDNA with **PA1**. (b) Diagram of the binding interaction of **PA1** (black) with dsDNA (blue). (c) Table showing the oligodeoxyribonucleotide (ODN) sequences studied and abbreviations used in the text. Boldfaced letters correspond to the binding site of **PA1**.

The inherent challenge in such a study lies in the fact that DNA duplexes are dynamic over a wide range of time and length scales, which enables the propagation of local conformational changes throughout the duplex.^17–20^ Of the spectroscopic methods used to explore dsDNA dynamics, temperature (T)-jump time-resolved infrared (IR) spectroscopy has emerged as a powerful tool to gain insight into structural changes following ultrafast (ns) thermal perturbation with base-pair resolution.^19,21–26^ In particular, *T*-jump IR probing of the recognition of dsDNA sequences by small molecules revealed that melting dynamics are slowed by a factor of 8 upon ligand binding.^21^ Minor groove ligand binding to dsDNA also reduced end-fraying dynamics of base-pairs adjacent to the binding site.^27^ In this context, end fraying serves as a good proxy for measuring remote changes in the dynamic behavior of the double helix caused by ligand binding, but strand separation is also a fundamental element of transcription and replication. At present, however, a quantitative experimental understanding of the distance dependence of the impact of binding upon the double helix is required to benchmark models of DNA dynamics and guide ligand design processes.^3,4^

Our approach used a series of dsDNA oligodeoxyribonucleotide (ODN) sequences (**8-ODN** to **14-ODN**, Figure 1(c)) that incorporate a core **PA1** (Figure 1) binding site 5′-ATGTACAC-3′ (underlined) extended by successive additions of A-T base-pairs in the 3′ direction up to a maximum of 14 base-pairs (Figure 1(c)). Our results show that the minor groove ligand **PA1** suppresses end-fraying dynamics and delays double-strand melting. Quantitative analysis of the sequence length dependence of the end fraying suppression revealed that the allosteric effect follows an exponential distance profile with a propagation length of some 30 base-pairs, showing that the PA ligand has a considerable effect on the long-range dynamics of dsDNA.

PA•DNA complexes were formed by titration of **PA1** into a solution of dsDNA. Monitoring by ^1^H NMR spectroscopy ensured formation of a 1:1 complex (for **PA1** preparation, characterization and complex formation, see Supporting Information Scheme S1, Figures S1–S9 and Table S1). IR absorption spectra were measured using a Bruker Vertex 70 Fourier transform (FT)-IR spectrometer. *T*-jump IR spectroscopy was performed using a ns-duration *T*-jump pump pulse tuned to the O–D stretching vibration of the D_2_O solvent to deliver a 12 K increase in temperature.^28^ A time-delayed IR probe pulse resonant with the base vibrational modes of DNA was used to monitor the subsequent temporal evolution of the system.^28^ Full details of sample preparation and spectroscopy experiments are provided in the Supporting Information.

Melting curves for the series of ODNs with and without **PA1** bound were obtained from temperature-dependent FTIR spectra by plotting the absorbance of the band due to the guanine ring (G_R_) vibrational mode at 1575 cm^–1^ as a function of temperature (Figure 2(a,b), Figure S10). The G_R_ band increases in intensity upon dsDNA melting, primarily due to loss of base stacking.^19,29,30^

**Figure 2:**
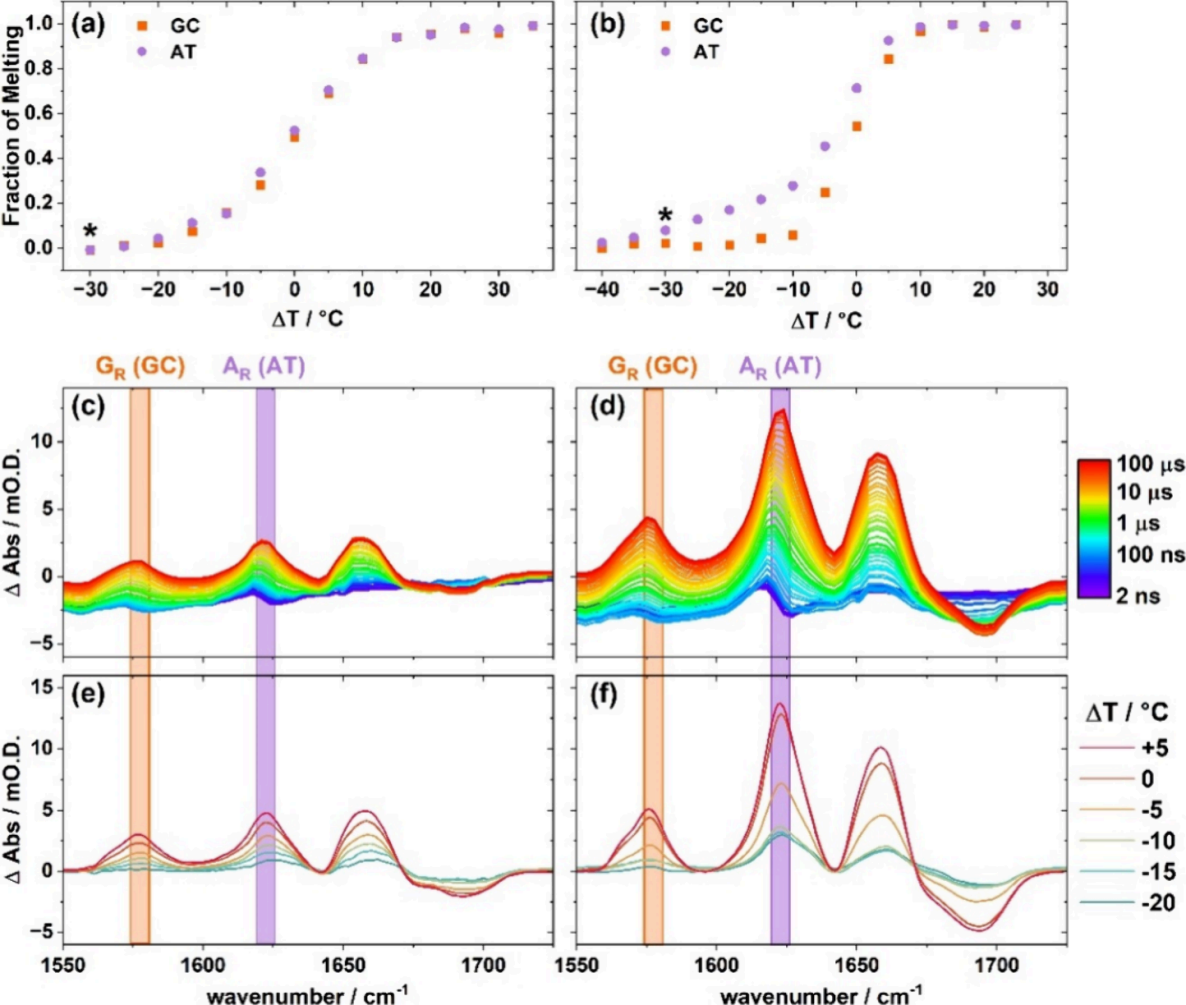
Melting curves derived from the change in absorbance of the G_R_ (orange) and A_R_ (purple) bands of **8-ODN** (a) and **14-ODN** (b) as a function of Δ*T* (Δ*T* = *T*_0_ – *T*_m_, where *T*_0_ is the equilibrium temperature of an IR absorption measurement or the starting temperature of a *T*-jump measurement and *T*_m_ is the melting temperature). All data sets are normalized to the results of fitting the experimental data with a two-state sigmoidal function show the fraction of melting. More detail is given in the Supporting Information (Figure S10). Asterisk marks the initial temperature for the spectra in Figure 3. *T*-jump IR spectra of **8-ODN** (c) and **14-ODN** (d) obtained as a function of *T*-jump pump–probe delay time from 2 ns (blue) to 100 μs (red), at Δ*T* = 0 °C (see text). ΔAbs is given in units of optical density (O.D.) which are equivalent to absorbance units. Colored bands highlight the position of the G_R_ (orange) and A_R_ (purple) bands. Difference FTIR spectra representing the effect of an increase in temperature of 10 °C on **8-ODN** (e) and **14-ODN** (f) as a function of Δ*T*.

Binding of **PA1** increased the melting temperature (*T*_m_) of the complexes by 27–33 °C relative to the unbound **n-ODN**. Since each complex has a different *T*_m_ (Table S2), subsequent data is presented as a function of Δ*T* = *T*_0_ – *T*_m_, where *T*_0_ is the equilibrium temperature of an IR absorption measurement or the starting temperature of a *T*-jump measurement.

*T*-jump-IR experiments performed on **8**- and **14-ODN** near *T*_m_ (Δ*T* = 0 °C: Figure 2(c,d)) showed a characteristic band pattern associated with dsDNA melting.^19,21,27,31^ The data are presented as *T*-jump pump_on_ – pump_off_ difference spectra so that positive features indicate bands that increased in intensity following the *T*-jump and *vice versa*. In addition to changes in the 1650–1700 cm^–1^ region, which contains contributions from a number of overlapping base vibrational modes, bands at 1575 and 1625 cm^–1^ were observed to increase in intensity (Figure 2(c,d), orange and purple bars). The former band arises from the G_R_ mode, while the latter is assigned to an equivalent mode of adenine (A_R_), which also increases in intensity upon dsDNA melting.^29^ The absorbances of these bands provide a quantitative measure of dsDNA disruption and continue to rise until a *T*-jump pump–probe delay time of around 25 μs before decaying to the baseline as the sample cools on ms-time scales (Figures S11 and S12). Comparison of the *T*-jump spectra with FTIR difference spectra mimicking a similar rise in temperature (Figure 2(e,f)) show a near identical band profile, confirming assignment of the spectral changes to dsDNA melting.

Examining the temporal profile of the increase in intensity of the A_R_ mode following the *T*-jump showed it to be well-represented by a triexponential function producing three time scales in the regions τ_1_: 10–20 ns, τ_2_: 100–200 ns and τ_3_: 4–5 μs (Figure 3(a)). These observations are in line with previous studies of dsDNA melting, allowing assignment of the time scales to the initial thermal response of the sample, end-fraying and duplex melting, respectively.^19,22–24,27,32^

**Figure 3:**
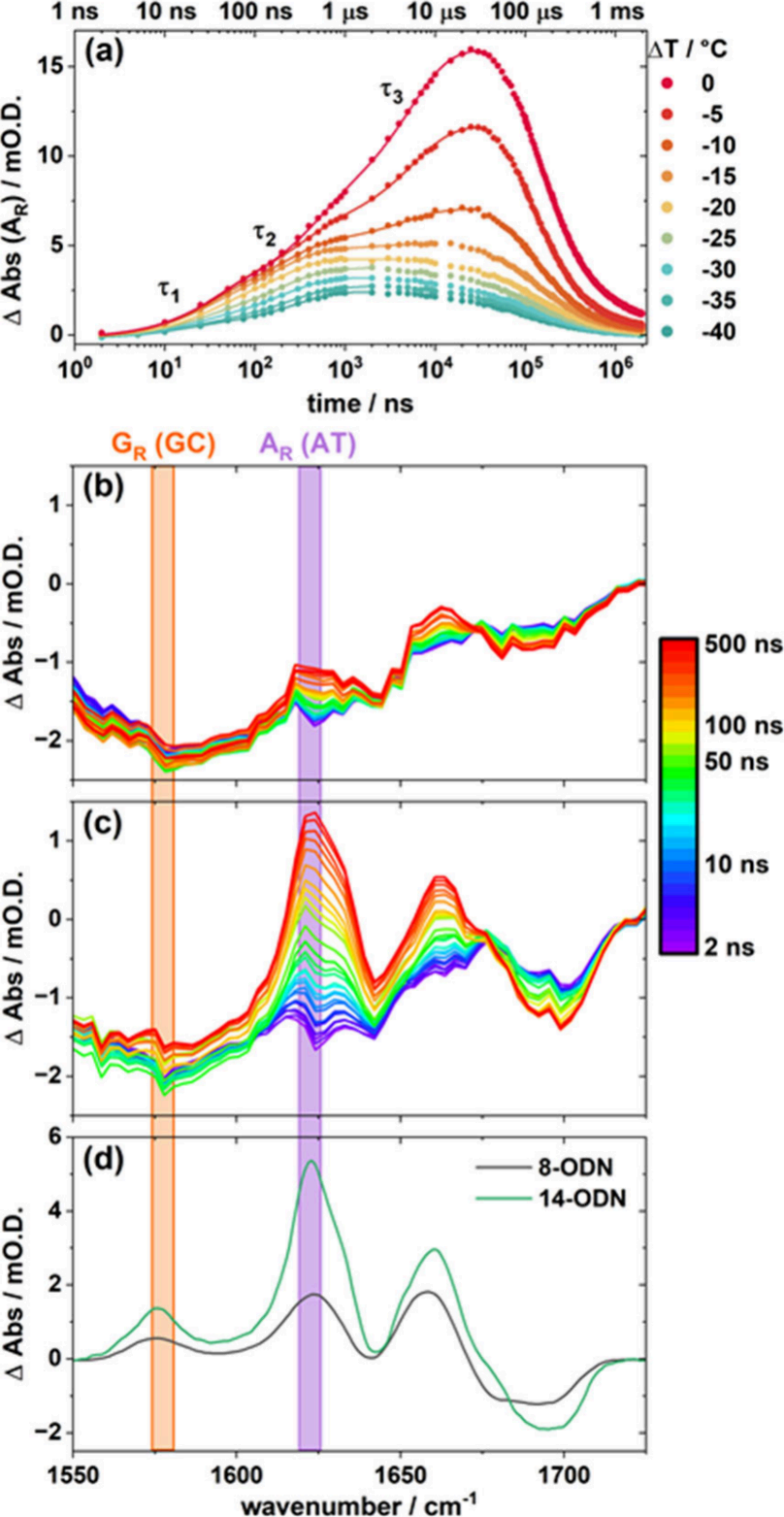
(a) *T*-jump dynamics of the A_R_ band of **14-ODN** as a function of Δ*T* (points) along with the results of triexponential fitting as described in the text. The lifetimes indicated are for the initial thermal response (τ_1_), end-fraying (τ_2_) and melting (τ_3_). *T*-jump IR difference spectra of **8-ODN** (b) and **14-ODN** (c) obtained as a function of *T*-jump pump–probe delay time from 2 to 500 ns, at Δ*T* = −30 °C. Equilibrium difference IR absorption spectra (d) representing a jump in temperature of 10 °C at Δ*T* = −30 °C for **8-ODN** (black) and **14-ODN** (green).

To focus on the near-equilibrium dynamics of the helix, which are most relevant to the question of allosteric communication, *T*-jump IR difference spectra were obtained at an initial temperature far from the melting transition (Figure 3(b,c)). At Δ*T* = −30 °C, increases in A_R_ mode (purple) intensity were observed for both **8-ODN** (Figure 3(b)) and **14-ODN** (Figure 3(c)) sequences, but with no accompanying change in the G_R_ mode (orange). The lack of change in the G_R_ mode is consistent with no duplex melting occurring this far from *T*_m_ (Figure 2(a,b)). This is due to the location of the majority of the G-C base-pairs in the center of the dsDNA sequences and the relative resistance of terminal G-C pairs to end fraying as a result of the need to break three H-bonds.^16^ The observed change in the A_R_ mode intensity at this Δ*T* is therefore assigned to perturbation of the A-T base-pairs, including end-fraying at the 5′ end of **8-ODN** and at both the 3′- and 5′-ends of **14-ODN**. The results are in good agreement with difference FTIR spectra obtained over a similar temperature range (Figure 3(d)), while the time scales of the changes in the A_R_ band, which occur around ∼100–200 ns (Figure 3(a)) are also consistent with previous observations of end-fraying.^19,21,22,27,31^ Fitting the temporal dynamics as a function of *T*_0_ allowed an Eyring analysis to be performed (Figure S13, Table S3), producing an activation enthalpy of 18–25 kJ mol^–1^, approximately equivalent to the loss of two hydrogen bonds, as would be expected for an A-T base-pair.^33,34^

Comparing the end fraying behavior of **8**- and **14-ODN** (Figure 3), it is noticeable that the increase in the A_R_ mode of **14-ODN** is greater than that observed for **8-ODN**. Given that there are different numbers of base-pairs in these two sequences, it is important to consider these changes quantitatively by comparing them to the overall increase in the A_R_ band intensity that occurs upon full melting of the respective dsDNA structure, which is available from the FTIR spectrum (Figure 2). In the case of **14-ODN**, at a temperature of Δ*T* = −20 °C, the approximate end point for a *T*-jump beginning at Δ*T* = −30 °C, the FTIR-derived A_R_ band intensity is 17% of the maximum melting signal, with an increase of ∼9% occurring over the 10 °C window. As there are 11 A-T base-pairs in the **14-ODN** sequence (Figure 1), this equates to the equivalent of approximately two base-pairs per dsDNA having frayed by Δ*T* = −20 °C. In the case of **8-ODN**, the increase between Δ*T* = −30 °C and Δ*T* = −20 °C is around 4% of the total, with the equivalent of 5% of A-T pairs having frayed by Δ*T* = −20 °C. This equates to less than one base-pair per duplex over the ensemble (Table S4). Overall, this shows that **14-ODN** is more prone to end-fraying than **8-ODN**.

Considering end-fraying as a function of Δ*T* for **8**- and **14-ODN** (Figure 4, red bars) reveals a similar pattern, with **14-ODN** giving rise to a greater absolute change in the intensity of the A_R_ band than **8-ODN** at all values of Δ*T*. However, for a given sequence, the contribution to the A_R_ band amplitude assigned to end fraying (that occurring between 2 and 100 ns, red bars),^27^ remains approximately constant with Δ*T*. This is in contrast to the contribution to the A_R_ band intensity occurring between 100 ns to 10 μs (blue bars), which is due to duplex melting. The latter increases dramatically as *T*_m_ is approached in line with the sigmoidal melting behavior of the sequence. This is consistent with previous observations showing that the fluctuations of the strand leading to end-fraying are separable from the melting behavior.^27^

**Figure 4:**
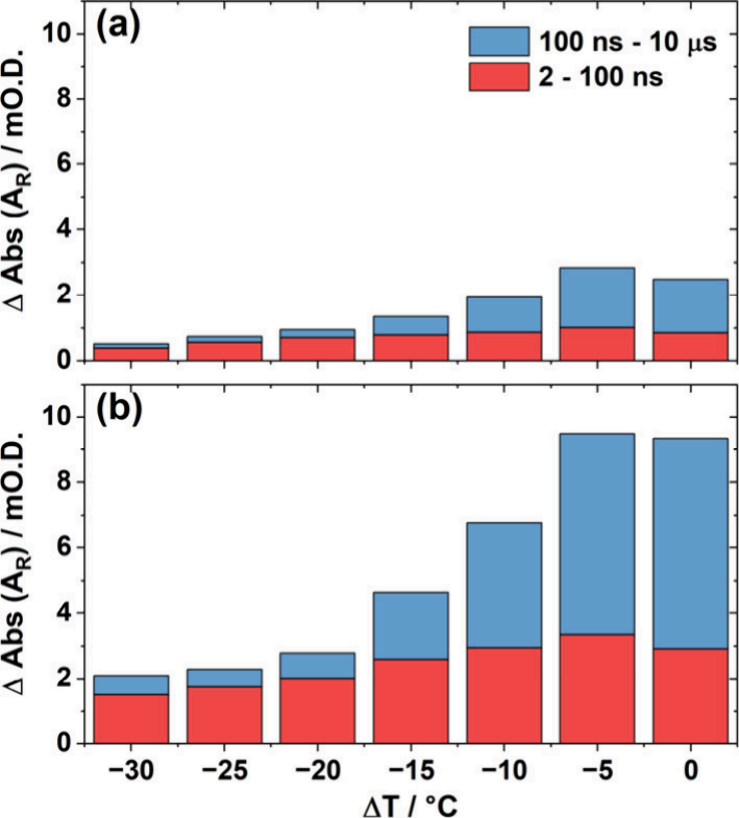
Comparison of the measured increases in A_R_ band absorbance as a function of Δ*T* for the *T*-jump IR experiments of **8-ODN** (a) and **14-ODN** (b) between 2 and 100 ns (red) and between 100 ns and 10 μs (blue).

Extending the study to include the **10**- and **12-ODN** sequences shows intermediate behavior relative to **8**- and **14-ODN** in terms of the magnitude of the changes in the A_R_ band due to end fraying. The equivalent data for these sequences are shown in Figures S14 and 15 and Table S4.

Having established the dynamic behavior of free duplexes we progress to comparisons with data obtained on **n-ODN•PA1** complexes to establish the dynamic impact of ligand binding. The results of *T*-jump spectroscopy measurements on **8-ODN•PA1** and **14-ODN•PA1** are shown in Figure 5 alongside the equivalent data for the ODN in the absence of **PA1**.

**Figure 5:**
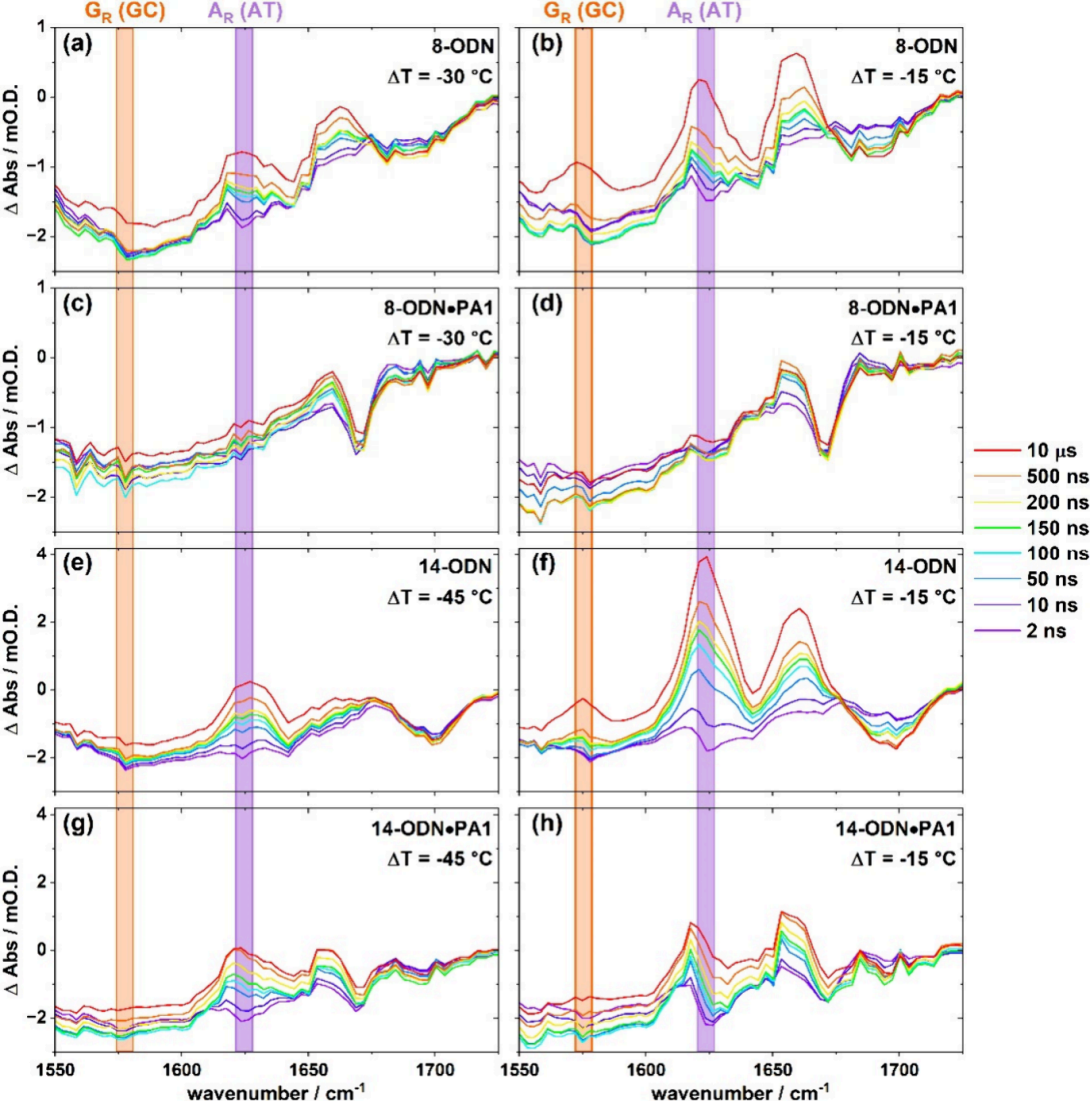
*T*-jump IR spectra of **8-ODN** (a and b), **8-ODN•PA1** (c and d), **14-ODN** (e and f), **14-ODN•PA1** (g and h) obtained as a function of *T*-jump-probe delay time from 2 ns to 10 μs. Spectra were obtained at Δ*T* values of −30 °C (a and c), −15 °C (b, d, f and h) and −45 °C (e and g).

At large negative Δ*T* values, far from the melting temperature, we observe the characteristic A_R_ band appearing within 100 ns of the *T*-jump pulse in both **8**- and **14-ODN** (Figure 5(a,e), purple), indicative of end-fraying. In the presence of the ligand, however, no equivalent band is present in the data obtained for **8-ODN•PA1** (Figure 5(c)). Indeed, the only signal observed across all pump–probe delay times is a negative peak at 1670 cm^–1^. This corresponds to the ν­(C=O), carboxylate stretching mode of trifluoroacetic acid (TFA), which is present in trace amounts in all of the complexes as a result of its use in the purification process of **PA1**. This situation remained unchanged upon altering Δ*T* to −15 °C, closer to *T*_m_ (Figure 5(d)).

Comparing the *T*-jump data from **8-ODN•PA1** to the FTIR spectrum obtained under equilibrium conditions reveals a small increase in the A_R_ mode and a larger increase in the G_R_ mode in the latter (Figure S16). This shows that a small amount of melting does occur for **8-ODN•PA1** at this temperature on a longer time scale, which is not captured in the *T*-jump data. Furthermore, as *T*_0_ was moved progressively closer to *T*_m_, contributions from the G_R_ band appear in the *T*-jump-IR spectra of **8-ODN** at later *T*-jump-probe times (Figure 5(b), orange), but were not observed for the complex (Figure 5(d)). Taken together, the lack of contribution to the *T*-jump-IR spectra from the A_R_ and G_R_ modes of **8-ODN•PA1** across all *T*-jump-probe delays suggests that binding of **PA1** has significantly suppressed both the fast dynamics of **8-ODN**, reaching up to two base-pairs from the binding site, and delayed the melting behavior. These observations match those made using the Hoechst 33258 dye in different dsDNA sequences.^21,27^

It is interesting to note that, in addition to end-fraying and melting, there was also a lack of spectroscopic response of **8-ODN•PA1** compared to **8-ODN** at short pump–probe delay times between 10 and 50 ns (Figure 5(a–d), blue). On these time scales, changes appear in the free dsDNA spectrum at 1625 and 1660 cm^–1^, which are assigned to base vibrational modes of dsDNA responding to the initial change in temperature caused by the *T*-jump pulse. That no similar signature is observed for **8-ODN•PA1** is attributed to the location of the **PA1** ligand (*K*_d_ = 254 pM) in the minor groove of dsDNA.^35^ The strongly bound ligand displaces the solvating water backbone found in this groove and we hypothesize that the change in solvation environment restricts the response of the DNA vibrational modes.^36,37^ By contrast, the TFA contaminant is not bound to the complex and so reports the change in temperature through a shift in its carboxyl stretching vibrational mode (1670 cm^–1^) upon rapid heating.

Upon increasing the length of the dsDNA sequence, a response from the A_R_ mode was observed in the *T*-jump spectra of **14-ODN•PA1** at large, negative values of Δ*T* (Figure 5(g)), in contrast to observations from **8-ODN•PA1**. Although this appears similar to observations made on the unbound **14-ODN** duplex (Figure 5(e)), comparing the absolute values to the equilibrium IR data shows that, for **14-ODN**, the change in absorbance of the A_R_ band in the *T*-jump data at a delay time of 100 ns is equivalent to 44% of the change in absorbance observed under equilibrium conditions. For **14-ODN•PA1**, this proportion is just 13%, indicative of **PA1** binding to **14-ODN** decreasing end-fraying by two-thirds. The melting dynamics are also affected upon ligand binding as the growth of the G_R_ absorbance occurs at later times in **14-ODN•PA1** compared to **14-ODN** (Figure S17). Therefore, the presence of the **PA1** ligand also alters the fast dynamics of **14-ODN** at large Δ*T* values, although to a lesser degree than for **8-ODN**. Taken collectively, this data shows that the suppressive effect of minor groove recognition by **PA1** propagates to the end of the 14-mer sequence. The reduction of the dynamic suppression effect at the end of the **14-ODN** sequence compared to that observed for **8-ODN** is consistent with a general reduction in the allosteric effect with distance, as proposed by models.^3,38,39^

Unlike the **8-ODN•PA1** complex, an A_R_ mode response is observed for **14-ODN•PA1** even at the shortest of *T*-jump-probe delay times (Figure 5(g)). This is consistent with our earlier hypothesis in that, as the sequence length increases, water can now occupy the minor groove in the section of the dsDNA located away from the binding site, solvating the A-T nucleobases.^36,37^ The resulting change in solvation leads to the small response of the A_R_ mode seen at early delay times. We note that the spectral position of this thermal response occurs at a slightly lower wavenumber than the end fraying response, as would be expected for two different processes. This observation is more apparent at smaller negative values of Δ*T*. Specifically, for **14-ODN**, the magnitude of the A_R_ band increases as Δ*T* is changed to −15 °C (Figure 5(f)), but for **14-ODN•PA1** (Figure 5(h)) the signal in the A_R_ region consists of a sharp positive and negative peak at early pump–probe delays (2–50 ns) that evolves into a single, broader, peak with time. This spectral evolution is assigned to the effect of two competing line shapes described above: the “positive–negative” heating response caused by a shift in the A_R_ band due to heating-induced modification of the solvation environment of dsDNA, which is replaced by the Gaussian A_R_ mode centered at 1625 cm^–1^ due to end fraying. Due to its size, the heating response masks the actual change in absorbance of the A_R_ band due to end-fraying at Δ*T* = −15 °C while also causing a small offset of the A_R_ peak center in the complex.

Reconciling the aforementioned results with analysis of the **10**- and **12-ODN** sequences (Figures S18 and S19, Table S5) allows evaluation of the impact of **PA1** binding as a function of dsDNA length. Figure 6 shows the degree of end-fraying (defined as ΔAbs­(A_R_) at 100 ns/ΔAbs­(A_R_) under equilibrium conditions) for each sequence length and the respective **PA1** complex at Δ*T* = −40 °C. As expected, increasing the DNA sequence length results in an increase in the degree of end-fraying in free DNA. This is also true for the **n**-**ODN•PA1** complexes, although the trend is flatter such that we can infer that there is a large difference between dsDNA and dsDNA•PA for all sequence lengths. It has previously been shown that the finite propagation length of allosteric binding along DNA, ξ, can be estimated from the decrease in the change in absorbance of a vibrational mode upon ligand binding.^27^ Examining the decrease in the degree of end-fraying with sequence length, which is given by the percentage reduction in the degree of end-fraying for a given sequence due to the presence of **PA1** (Figure 6, red symbols), shows that the suppressive effect of **PA1** on end-fraying is greater closer to the binding site. Fitting to a single exponential function produces an allosteric propagation length of 32 base-pairs (Equation S1 and Figure S20).^27^ Thus, **PA1** binding to target binding sites across all of the dsDNA sequences explored in this study results in suppression of the fast dynamics of dsDNA, the degree of which decreases exponentially with sequence length. For completeness, measurements on a 14-mer dsDNA sequence with the AT base-pairs added to the 5′ direction relative to the binding site and its **PA1** complex were also performed (Figure S21), showing that suppression of end-fraying by PA1 occurs equally in both the 5′ and 3′ directions.

**Figure 6:**
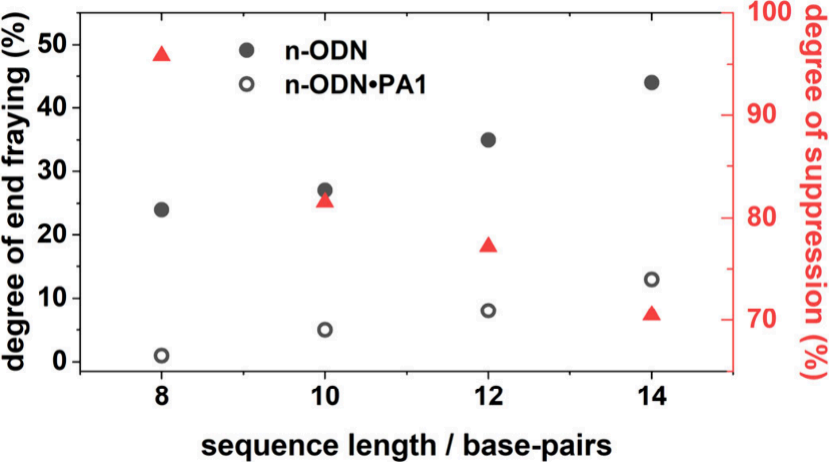
Degree of end-fraying for **n-ODN** sequences and **n-ODN•PA1** complexes obtained from comparisons of the A_R_ mode intensity obtained via *T*-jump spectroscopy at 100 ns and Δ*T* = −40 °C to those obtained from FTIR. Expressing the change in the degree of end-fraying upon binding **PA1** as a percentage gives the degree of suppression with sequence length.

The observed dynamic suppression is notably larger in comparison to another minor groove binder Hoechst 33258, which produced an estimated allosteric propagation length of 5 base-pairs.^27^ The two systems differ considerably in the size and shape of the ligand and this aspect would benefit from further study.^40,41^ From a biological perspective, our results show that the allosteric effect of PA binding could influence interactions many base pairs along the dsDNA sequence. More specifically, the androgen response element, on which the binding site used in this study is modeled, features a second GWWC binding site just 5 base-pairs away and which would be within reach of the dynamic effect arising from binding.

In conclusion, we have demonstrated that dsDNA recognition by a minor groove binding PA allosterically influences the long-range dsDNA dynamics, suppressing both end-fraying and double-strand melting. This effect persists in a manner consistent with a propagation length of 30 base-pairs and highlights a potential mechanism for remote communication between binding sites which could be exploited when designing synthetic ligands to modulate transcription.

## Supplementary Material



## Notes

Sample preparation and additional experimental details; additional spectroscopic data: temperature-dependent FT-IR melting curves of all samples, *T*-jump dynamics for all samples (PDF)
