# Supplementary material for: Long-Range Allosteric Modulation of DNA Duplex Dynamics Induced by Pyrrole-Imidazole Polyamide Binding
Source: J Phys Chem Lett. 2025 Jul 28;16(31):7875–82. doi: 10.1021/acs.jpclett.5c01542 (PMC12337142; doi:10.1021/acs.jpclett.5c01542)
Supplement: Supplementary file 1 [file jz5c01542_si_001.pdf]

Long-Range Allosteric Modulation of DNA Duplex Dynamics Induced by Pyrrole-Imidazole Polyamide Binding

Supporting Information

Sophie E.T. Kendall-Price,[1] Ryan J.O. Nichol,[2] Andrea Taladriz-Sender,[2] Ryan Phelps,[3] Partha Malakar,[3] Gregory M. Greetham,[3] Glenn A. Burley,[2,] Neil T. Hunt[1,]**

1) Department of Chemistry and York Biomedical Research Institute, University of York, Heslington, York, YO10 5DD, UK

2) Department of Pure and Applied Chemistry, University of Strathclyde, Glasgow, G1 1BX, UK

3) STFC Central Laser Facility, Research Complex at Harwell, Harwell Science and Innovation Campus, Didcot, OX11 0QX, UK

AUTHOR INFORMATION

Corresponding Author

* neil.hunt@york.ac.uk

* glenn.burley@strath.ac.uk

Table of Contents

1. Supplementary methods 3
1.1. Synthetic general procedures 3
1.2. Procedure for manual solid phase synthesis of polyamide (PA1) 3
1.3. Polyamide Characterisation (PA1) 6
1.4. Oligonucleotides used in the NMR and IR experiments. 8
1.5. Formation of PA1•n-ODN complexes 8
1.6. NMR titrations of PA1•n-ODN complexes 10
1.7. IR spectroscopy 14

1. **Supplementary methods**

1.1. **Synthetic general procedures**

All reagents and solvents were used as supplied from commercial sources and used without further purification unless otherwise stated. Solvents were all HPLC grade and were used without further purification, unless otherwise stated. Microwave-assisted reactions were carried out using a Biotage® Initiator +. Reactions were conducted at 75 °C for 30 minutes or one hour, depending on the substrate. NMR spectroscopy was carried out using a Bruker Avance Neo 600 MHz spectrometer. All chemical shifts (δ) were referenced to the deuterium lock and are reported in parts per million (ppm). All NMR data was processed using MestreNova software. Semi-preparative purification was carried out on Dionex Ultimate 3000 series instrument using a Kinetex 5μm C18 column (size 150 × 21.2 mm). Analytical HPLC traces were recorded on a Dionex Ultimate 3000 series HPLC using Aeris 2.6 μm PEPTIDE XB-C18 (size 250 × 4.6 mm). High Resolution Mass Spectrometry (HRMS) was performed on a ThermoScientific Exactive™ Plus Orbitrap Mass Spectrometer using electrospray ionization.

1.2. **Procedure for manual solid phase synthesis of polyamide (PA1)**

Polyamide **PA1** was prepared using Boc-β-Ala-PAM resin (0.5 mmol/g loading) purchased from Merk (Catalogue number 09846). The Fmoc protected pyrrole (CAS: 195387-29-2) and imidazole (CAS: 252206-28-3) building blocks were commercially available and purchased from Fujifilm.

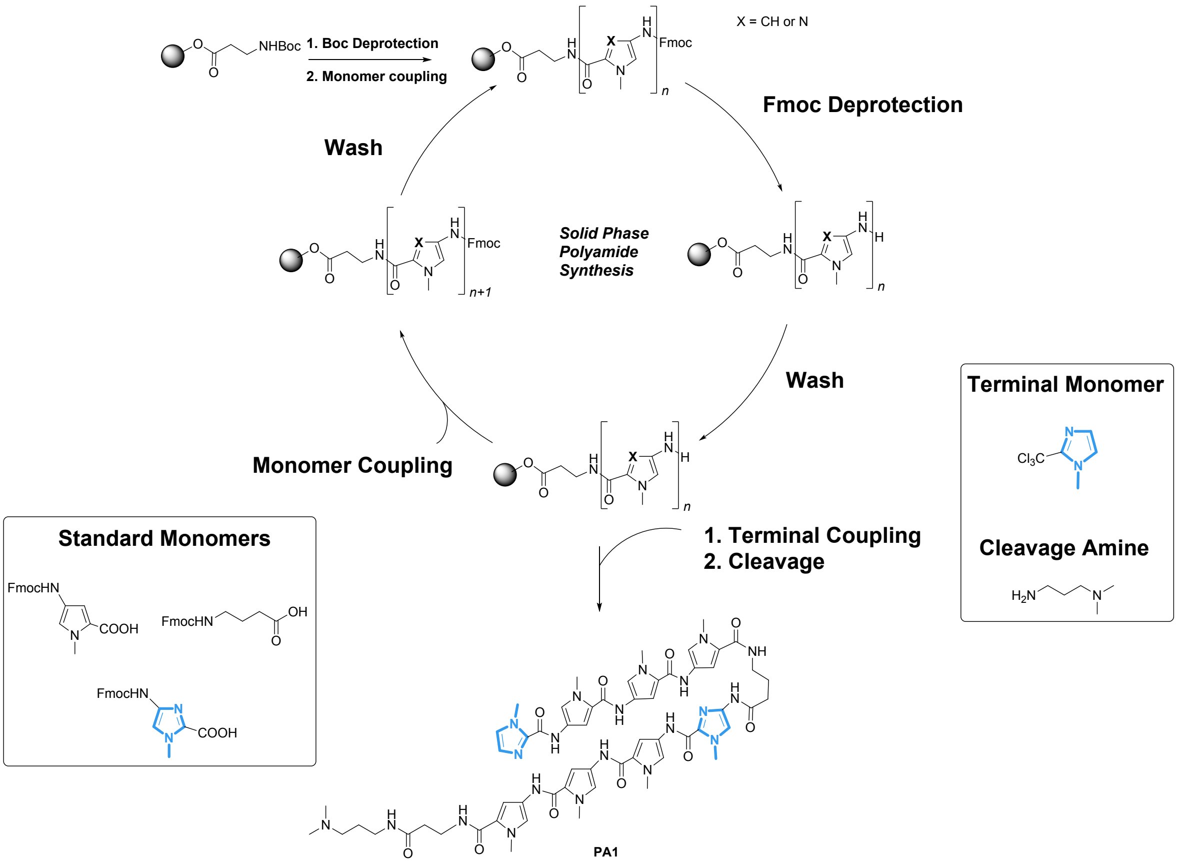

Scheme S1. Synthesis of polyamide **PA1**.

Resin preparation and deprotection of Boc group

The resin was washed using DMF (3 ×) and DCM (3 ×). Subsequently, the resin was swelled in DCM for 30 minutes. Next, the Boc group was removed upon treatment with a solution of TFA:Phenol:Water (TPW, 92.5/5/2.5) for 1 min followed by a wash with DCM (3 ×) and DMF (3 ×). This process was repeated twice with longer deprotection times (2 × 3 min). Then, the resin was basified using a solution of 50% DIPEA in DMF (v/v). The vessel was shaken for 3 minutes before was drained. The resin was washed using DCM, DMF and finally methanol. The washing cycle was repeated five times to ensure all side products and salts were removed from resin. The resin was then rinsed and dried using diethyl ether and transferred to a microwave vial for the following coupling step.

Coupling with Fmoc-Py-OH, Fmoc-Im-OH and Fmoc-β-Ala-OH

The building blocks (3 equiv.) were activated with HOBt (3 equiv.) and DIC (3 equiv.) in DMF (~ 1 mL) and shaken at room temperature for 10 min. The solution was added to the deprotected resin and then was heated to 75 °C for 30 mins using microwave irradiation. The resin was then filtered, washed (DMF × 3, DCM × 3) and taken forward to the next step.

Deprotection of Fmoc groups

After coupling, the Fmoc group was removed upon treatment with a solution 20% v/v of piperidine in DMF (2 × 10 min). Between each round of deprotection, the resin was washed with DMF (×3) and DCM (×3). The resin was rinsed and dried using diethyl ether and transferred to a microwave vial for the following coupling step.

Coupling with terminal Im-CCl_3

2,2,2-Trichloro-1-(1-methyl-*1H*-imidazol-2-yl)ethan-1-one (10 equiv.) was dissolved in DMF and DIPEA (500 µL) was added. The resulting solution was added to the deprotected resin and heated to 75 °C for 30 mins using microwave irradiation. The reaction was then shaken at room temperature overnight. The resin was filtered and washed (DMF × 3, DCM × 3).

Resin cleavage and purification

PA1 was cleaved from the solid support via nucleophilic displacement of the resin. The resin was suspended in N^1,N^1-dimethylpropane-1,3-diamine (~1 mL) and gently stirred at 70 °C overnight. The resin was filtered, rinsed with methanol and the filtrate concentrated under reduced pressure.

Crude **PA1** was purified through semipreparative RP-HPLC. A mixture of MeCN (0.1% TFA, v/v) and H_2O (0.1% TFA, v/v) was used as the eluent. The gradient used passed from 15% MeCN (0.1% TFA, v/v) to 35% MeCN (0.1% TFA, v/v) in 30 min at 17 mL/min. The desired

polyamide was isolated as TFA salt yielding yellow solid with purity ≥ 95%. Yields range from 5 – 20%.

1.3. **Polyamide Characterisation (PA1)**

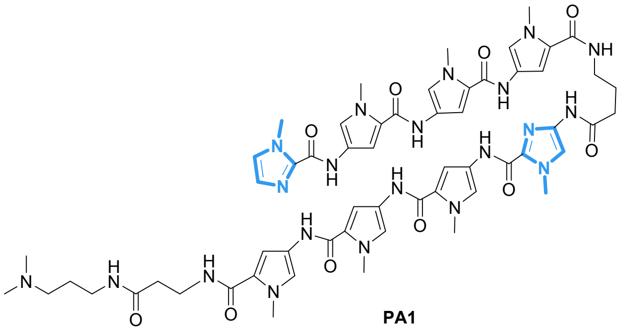

PA1

HRMS-ESI: exact mass calculated for $[M + H]^+$ $C_{58}H_{72}N_{21}O_{10}$ requires m/z 1222.5766, found $[M +H]^+$ m/z 1222.5791.

RP-HPLC purity (310 nm) = 98%. Rt = 13.6 minutes.

Analytical method: 5% MeCN (0.1% TFA, v/v) to 90% MeCN (0.1% TFA, v/v) in 30 min at 0.7 mL/min.

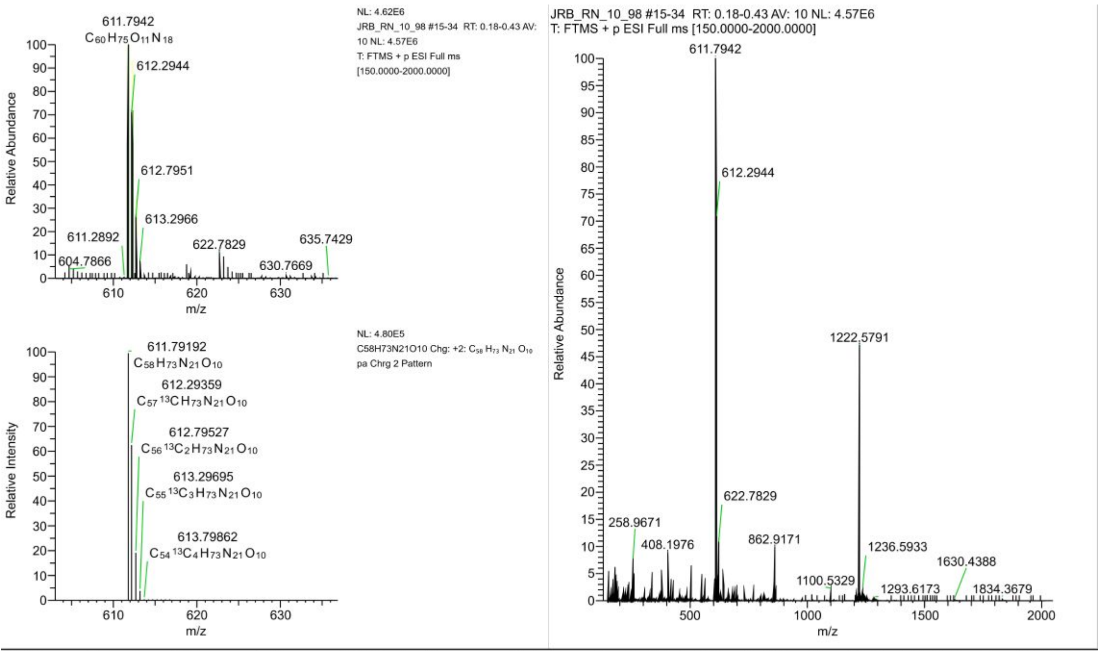

Peak Mass	Display For...	S Fit	RDB	Delta [ppm]	Theo. mass	Rank	Combined S...	# Matched I...	# Missed Iso.	MS Cov. [%]	Pattern Cov....	MSMS Matc...
611.7942	$C_{60}H_{75}O_{11}N_{18}$	72.3045333678446	32.50	2.72	611.79259	1	98.47	6	0	99.93	100	(Collection)
611.7942	$C_{58}H_{73}O_{10}N_{21}$	68.5204576272561	33.00	3.81	611.79192	2	98.28	6	0	99.93	100	(Collection)
611.7942	$C_{54}H_{75}O_{12}N_{22}$	6.89007608331472	28.50	-3.18	611.79619	3	46.92	6	0	49.14	50.19	(Collection)

Figure S1. High Resolution Mass Spectrum of **PA1**.

Analysis Report

**Sample Name:**	RN_10_98 B2	**Sample No.:**	3
**Sequence Name:**	RN_10_98 GS-PA purity check		
**Program Method:**	PolyamideAnalysis_5-95_30min_40min	**Injection vol.:**	20.0
**Quantitation Method:**	dna method	**Dilution Factor:**	1.0000
**Date Time Collected:**	11/7/2024 4:55 PM	**Sample Wt.:**	1.0000
**System Operator:**	Administrator	**Sample Amt.:**	1.0000

Peak No.	Component Name	Retention Time	Area mAU*min	Rel.Area %	Height mAU	Relative Height %
1	n.a.	13.33	0.943	1.23	19.410	1.42
2	n.a.	13.45	0.221	0.29	7.094	0.52
3	n.a.	13.58	75.180	97.82	1331.776	97.28
4	n.a.	13.80	0.510	0.66	10.666	0.78

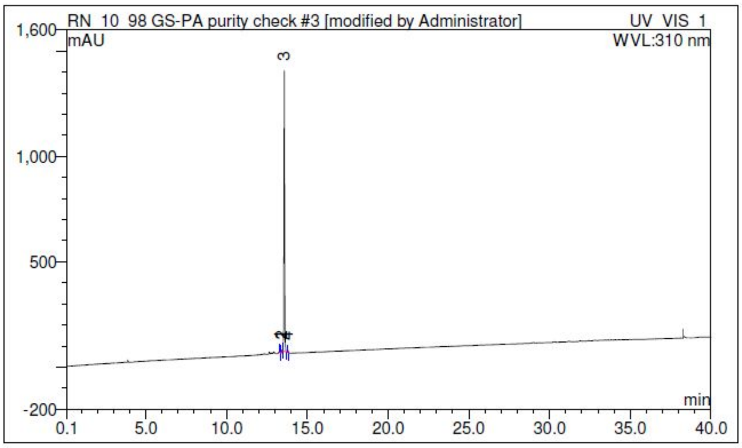

Figure S2. RP-HPLC trace of purified **PA1**.

1.4. **Oligonucleotides used in the NMR and IR experiments.**

Desalted lyophilized DNA oligonucleotide sequences were purchased from Eurofins and Eurogentec and used without further purification.

	ds DNA sequences
**8-ODN**	5′ ATGTACAC3′<br>3′ TACATGTG5′
**10-ODN**	5′ ATGTACACAA3′<br>3′ TACATGTGTT5′
**12-ODN**	5′ ATGTACACAATT3′<br>3′ TACATGTGTTAA5′
**14-ODN**	5′ ATGTACACAATTAT3′<br>3′ TACATGTGTTAATA5′

Table S1. DNA duplexes used in the paper

1.5. **Formation of PA1•n-ODN complexes**

DNA strands (~1 μmol) were hybridised with their complimentary sequence (~1 μmol) in 500 μL of 100 mM phosphate buffer in 90% H_2O and 10% D_2O (pH 7.0 – 7.2). Hybridisation was carried out with shaking at 25 °C for 30 min. Once hybridisation was complete, the **n-ODN** solution was transferred to an NMR tube. **PA1•n-ODN** complexes were formed starting with a **n-ODN** concentration of 2 mM and according to a previously published protocol.[1]

The formation of **PA1•n-ODN** complexes was monitored by ^{1}H NMR on a Bruker Neo 600 NMR spectrometer operating at a magnetic field strength of 14.1 Tesla (600.13 MHz for proton resonance) under TopSpin (version 4.5, Bruker, Reinstetten, Germany). The probe temperature was maintained at 298 K or 290 K depending on the **n-ODN** used.

A stock solution of **PA1** was prepared by dissolving > 1 μmol (1.5 – 2 mg) in 100 μL of MQ H_2O. The **PA1** stock solution was added in small aliquots to the buffered solution containing **n-ODN**. After each addition 1D ^{1}H NMR spectra were acquired until the end point of the

titration was reached as determined by inspection of the imino proton resonance region and PA aromatic region of the data (Figure S3). **PA1•n-ODN** complexes were then lyophilised.

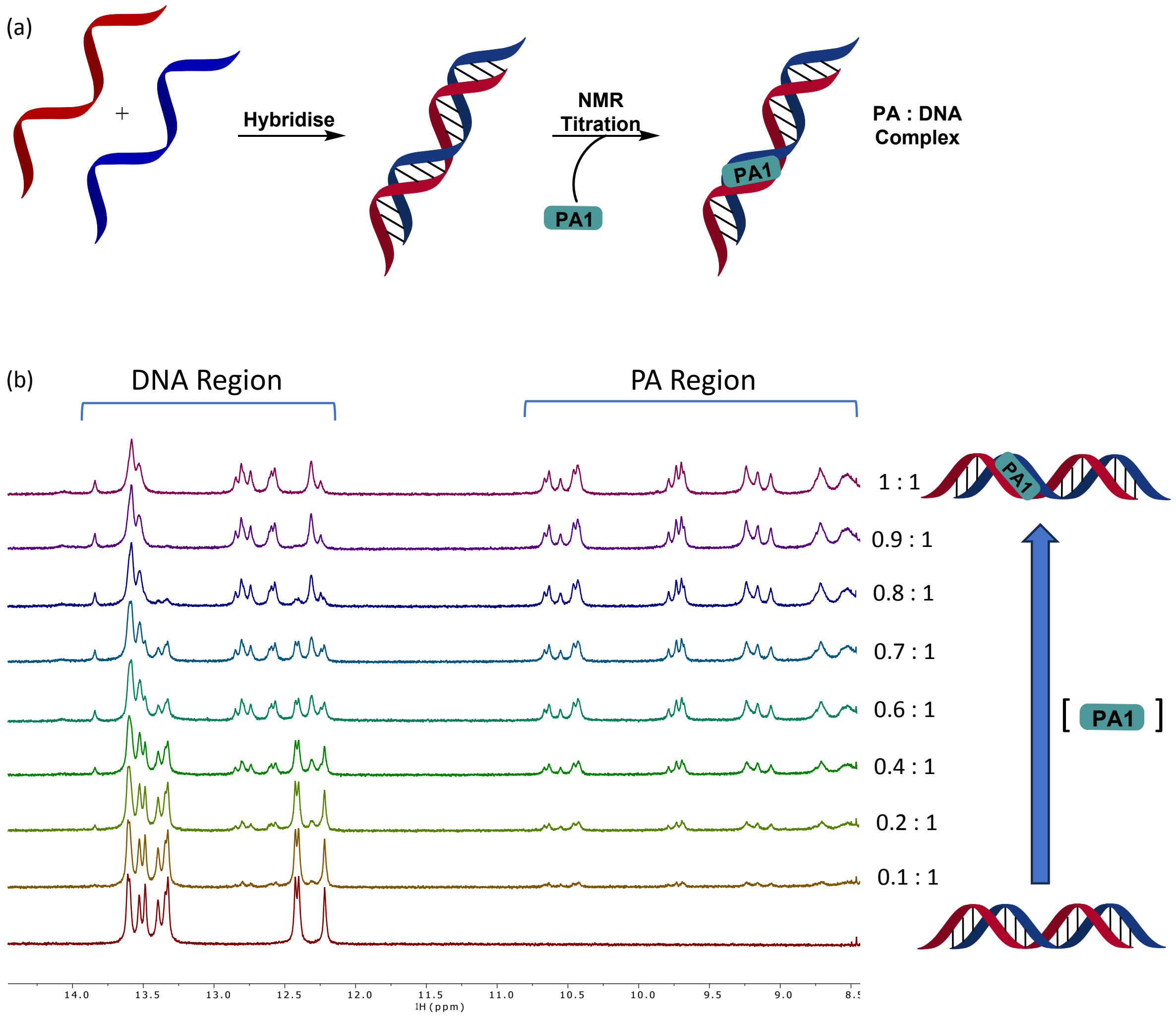

Figure S3. (a) Schematic representation of DNA hybridisation and titration with **PA1** to form **PA1•DNA** complexes.[1-2] (b) Expansion of diagnostic signals in the DNA exchangeable and PA backbone regions from ^{1}H NMR titrations of **PA1** with **14-ODN**. Spectra read from bottom to top, red (free DNA) to purple (**PA•DNA** complex).

1.6. **NMR titrations of PA1•n-ODN complexes**

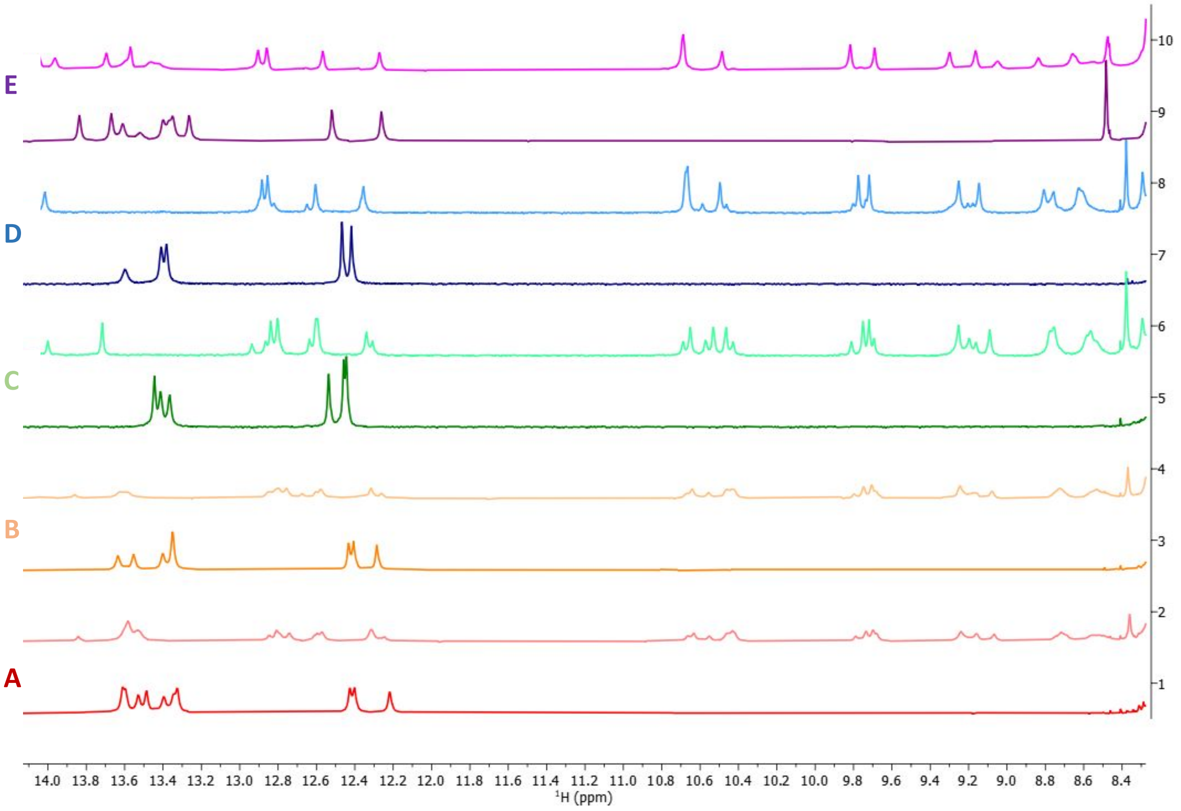

Figure S4. ^{1}H NMR Spectra expansion of DNA and PA regions showing the free **n-ODN** (odd spectra numbers) and the final point of the titrations of **PA1** with the corresponding **n-ODN** (even spectra numbers). Titration A, Dark red (1) – **14-ODN** and Light red (2) **PA1•14-ODN**. Titration B, Dark orange (3) **12-ODN** and Light orange (4) **PA1•12-ODN**. Titration C, Dark green (5) **10-ODN** and Light green (6) **PA1•10-ODN**. Titration D, Dark blue (7) **8-ODN** and Light blue (8) **PA1•8-ODN**. Titration E, Dark purple (9) **14-ODN-left fray** and Light purple (10) **PA1•.14-ODN-left fray.**

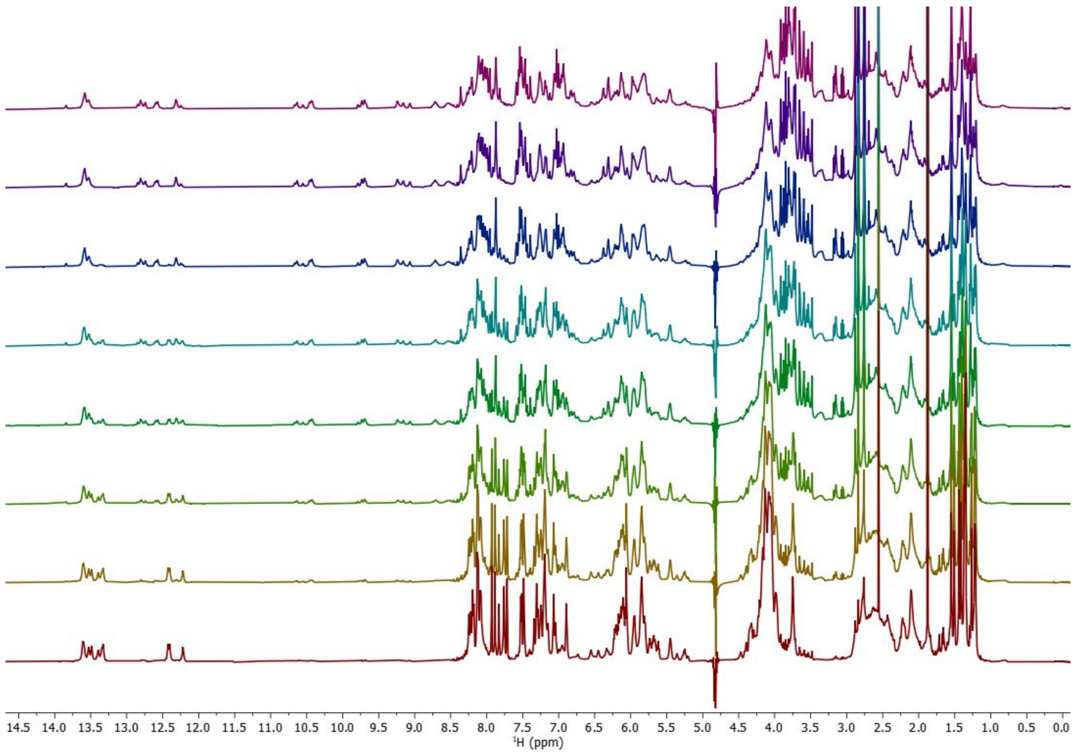

Figure S5. ^{1}H NMR titration of **PA1** with **14-ODN**. Spectra read from red (free **14-ODN**) to purple (**PA1•14-ODN** complex)

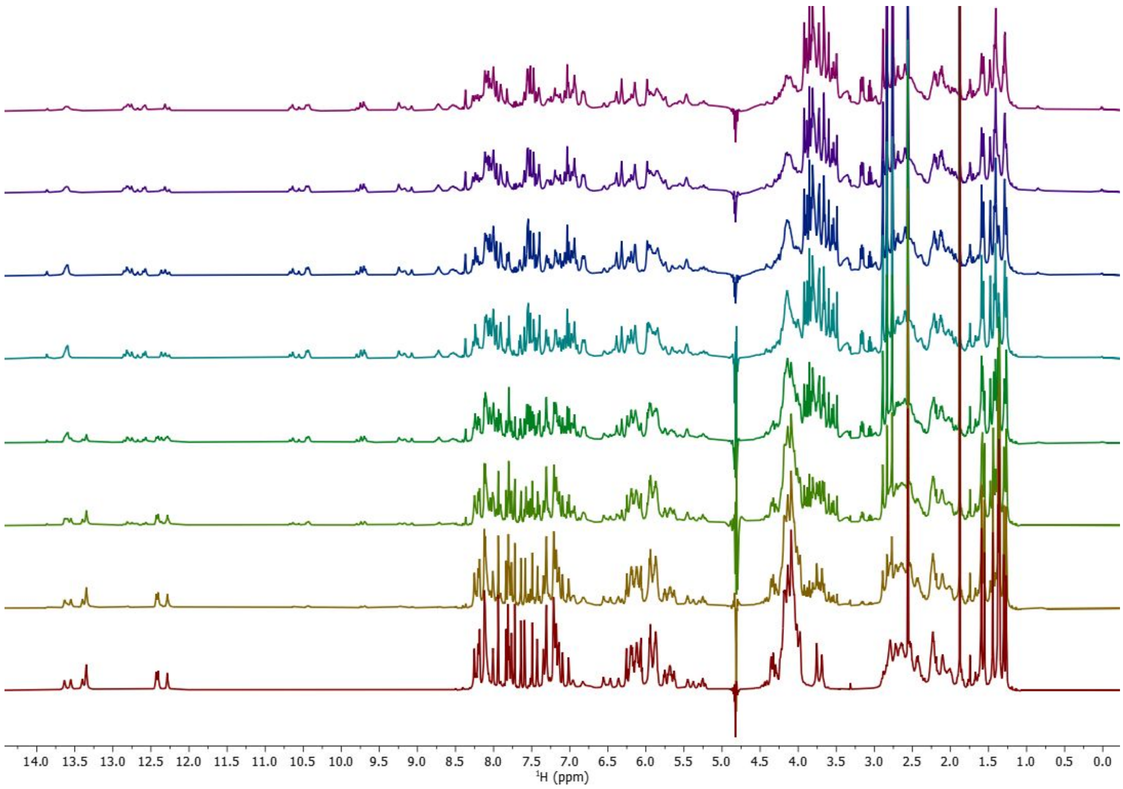

.

Figure S6. ^{1}H NMR titration of **PA1** with **12-ODN**. Spectra read from red (free **12-ODN**) to purple (**PA1•12-ODN** complex).

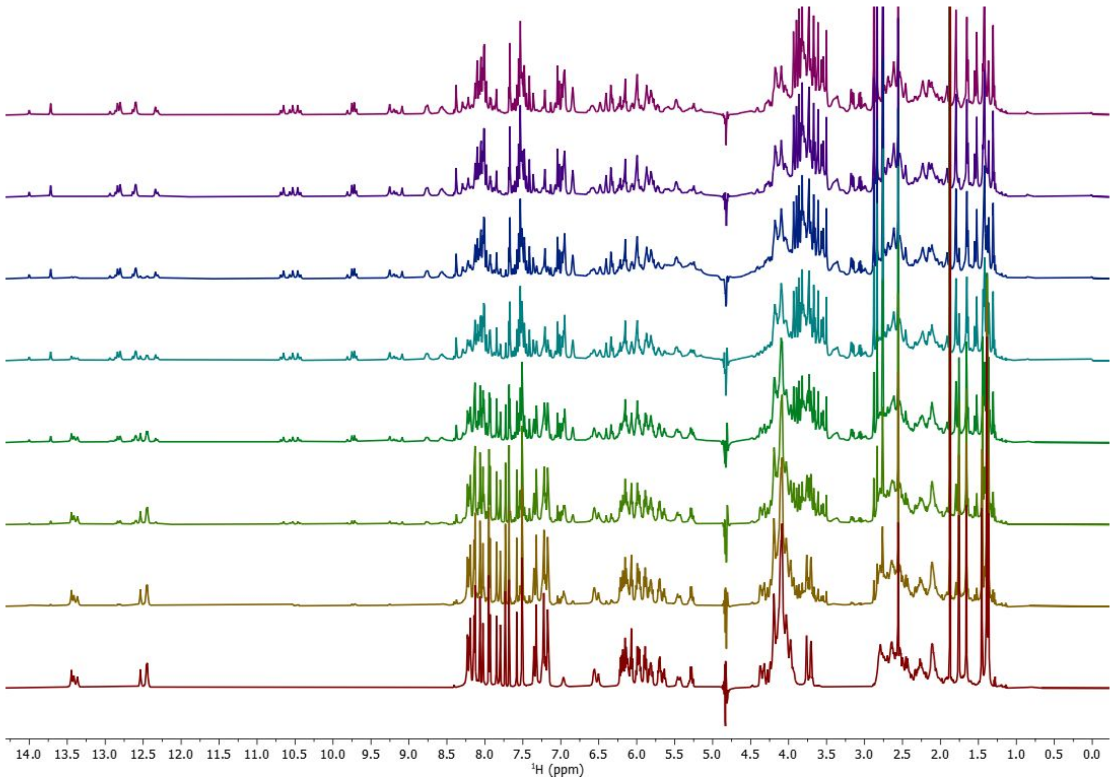

Figure S7. ^{1}H NMR titration of **PA1** with **10-ODN**. Spectra read from red (free **10-ODN**) to purple (**PA1•10-ODN** complex).

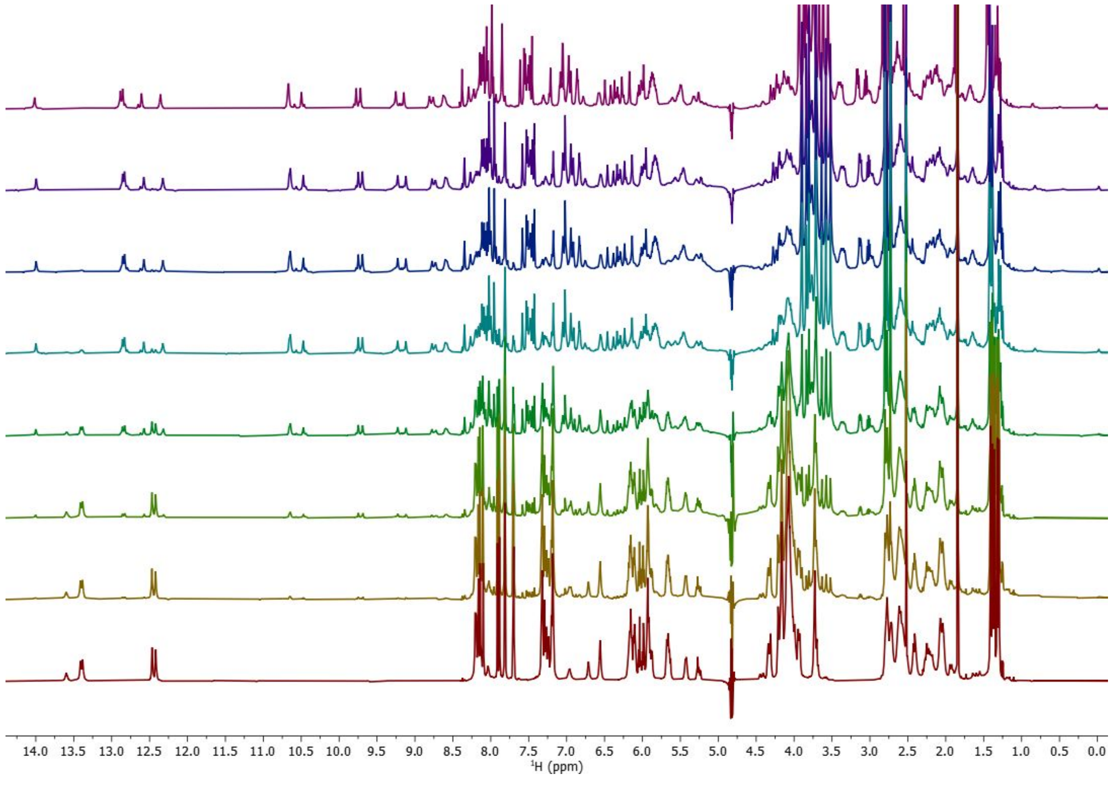

Figure S8. ^{1}H NMR titration of **PA1** with **8-ODN**. Spectra read from red (free **8-ODN**) to purple (**PA1•8-ODN** complex).

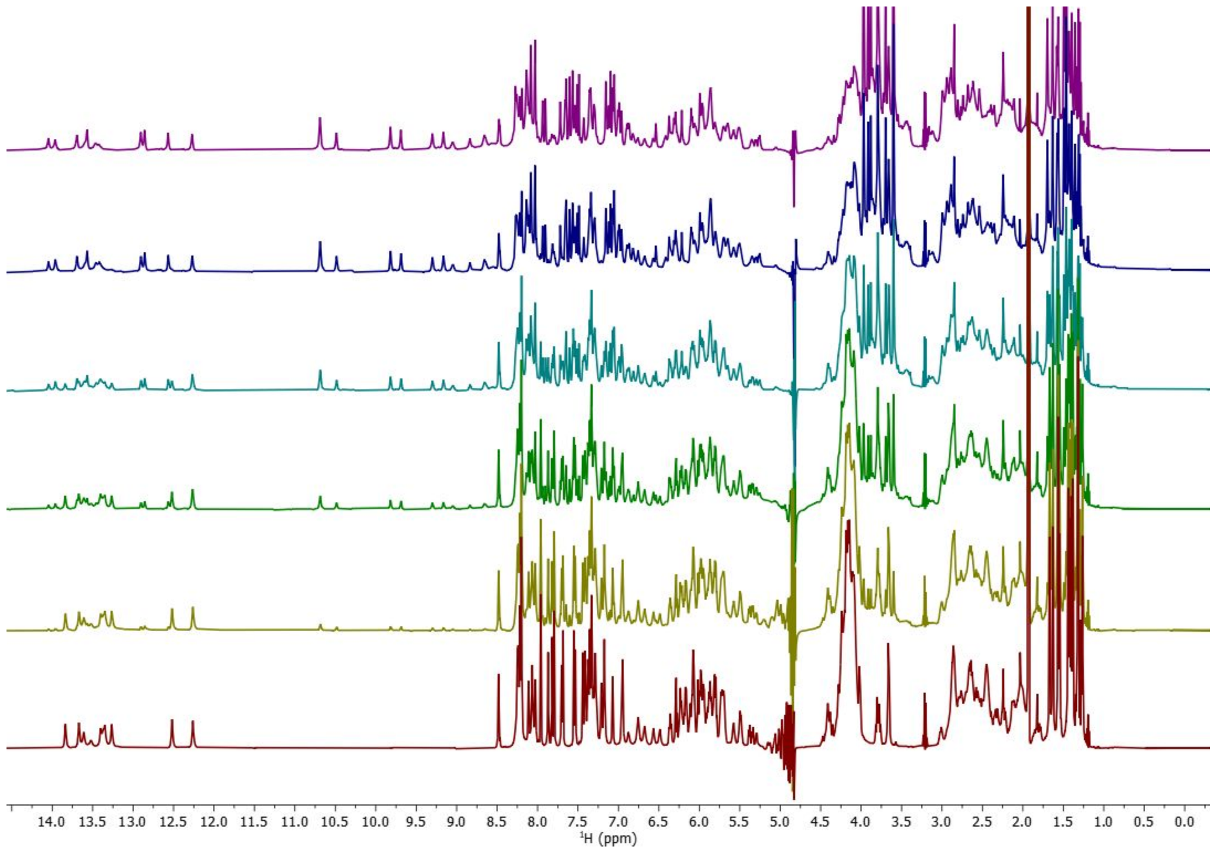

Figure S 9. ^{1}H NMR titration of **PA1** with **14-ODN-left fray**. Spectra read from red (free **14-ODN-left fray**) to purple (**PA1•14-ODN-left fray** complex).

1.7. **IR spectroscopy**

For all spectroscopy measurements, samples were prepared to a concentration of 10 mM in 100 mM deuterated phosphate buffer (100 mM NaCl, pD 7). A 15−20 μL aliquot of the solution was placed in a temperature-controlled cell (Harrick, ±1 °C) equipped with CaF_2 windows and a 12 μm path length.

IR absorption spectra were measured using a Bruker Vertex 70 Fourier transform (FT)-IR spectrometer with a resolution of 1 cm^{-1}. The T-jump measurements were performed using the STFC Central Laser Facility's ULTRA spectrometer, using a method that has been described in detail elsewhere.[3] Briefly, a 4 ns-duration (1 kHz, chopped to 500 Hz) T-jump pump pulse, tuned to the high frequency wing of the OD stretching vibrational band of the solvent was used to deliver a rapid increase in temperature from the initial value (T_0) set by the temperature-controlled sample cell. A time-delayed probe pulse (50 fs) tuned to coincide with the base stretching modes of the dsDNA sample near 1600 cm^{-1} was used to monitor the evolution of the sample following the T-jump. The T-jump pump-probe time delay was continuously variable from ns to 100 μs using a digital pulse delay generator, with the 100 μs to ms delays measured by subsequent probe pulses from with the probe laser, which operated at a repetition rate of 10 kHz. Calibration of the T-jump spectrometer with a solution of trifluoroacetic acid in D_2O, using previously reported methods,[3] established that the T-jump obtained was 12 °C.

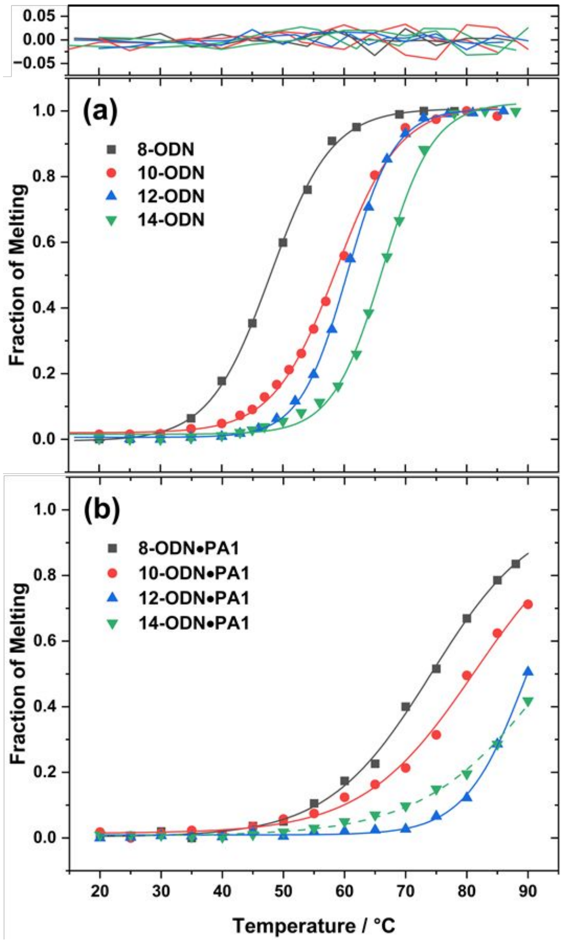

Figure S10. Melting curves for **n-ODN** sequences (a) and **n-ODN•PA1** complexes (b). These were derived from IR absorption spectroscopy experiments by plotting the intensity change of the G_R mode (1575 cm^{-1}) as a function of temperature. Prior to this, FTIR spectra were buffer subtracted and baseline corrected by subtracting the absorbance at 1750 cm^{-1}, which is in a region with no spectral features, from each data point. A linear temperature baseline extrapolated from the 20 – 30°C range where no melting occurs was subtracted from the melting curves followed by fitting the data using a two-state Boltzmann sigmoidal function ($y = \frac{A_1 - A_2}{1 + e^{(T-T_m)/dT}} + A_2$), where A_1 and A_2 represent the sigmoid minimum and maximum respectively; T_m represents the melting temperature and dT represents the slope constant. All datasets are normalized to A_2 to show fraction of melting ($\Delta Abs/\Delta Abs_{max}$). The residuals of the fits (top) show that the melting curves are well-represented by the two-state model. Dashed line in (b) signifies that the sigmoidal fit uses the estimated T_m value of 99 °C for **14-ODN•PA1**.

Table S2. Melting temperatures of each **n-ODN** sequence and **PA1•n-ODN** complex as calculated from fitting sigmoidal functions (Figure S10).

	**Melting temperature, T_m / °C**			
**n**	**8**	**10**	**12**	**14**
**n-ODN**	49 ± 0.2	59 ± 0.2	62 ± 0.1	66 ± 0.2
**PA1•n-ODN**	79 ± 0.9	86 ± 2.4	95 ± 2.0	–[1]

[1] T_m could not be obtained from the sigmoidal fit due to the high temperature but is estimated to be around 99°C based on the increase in T_m for the other **n-ODN** sequences upon binding of **PA1**.

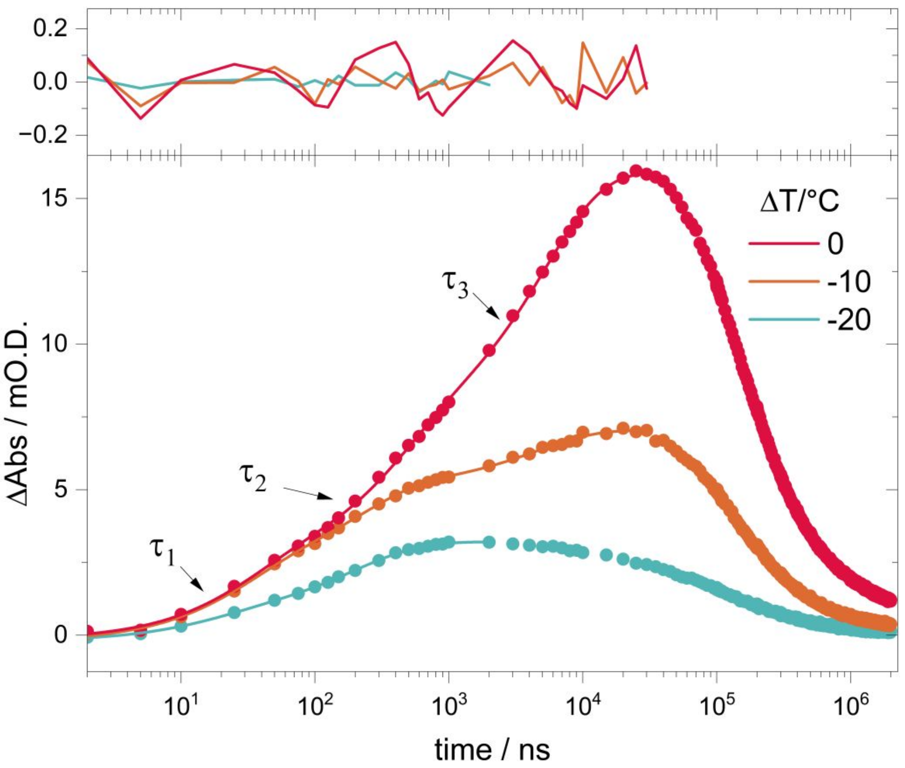

Figure S11. Temporal dependence of the A_R mode absorbance (dots, bottom) shown at three values of ΔT for **14-ODN.** The results of fitting the data with triple-exponential functions (lines, bottom) and residuals (top) are also shown. The lifetimes indicated (τ_n) are well-separated in terms of temporal region and assigned to the thermal response of the sample (τ_1), end-fraying (τ_2) and duplex melting (τ_3) respectively. Fitting was performed only to the absorbance maximum for each ΔT data set.

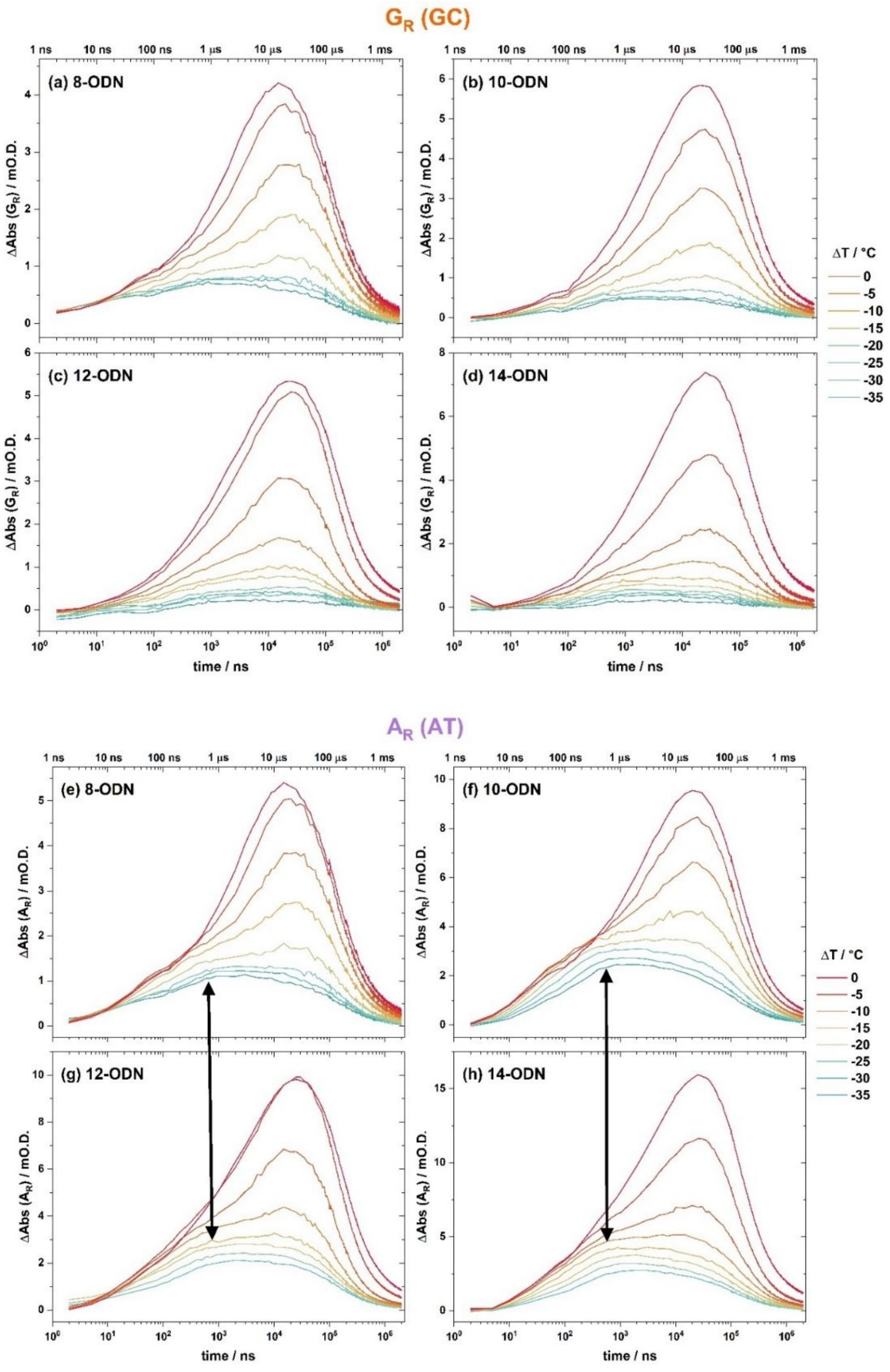

Figure S12. T-jump dynamics of the G_R (a-d) and A_R (e-h) mode in **n-ODN** as a function of ΔT. Data are shown from a ΔT value of −35 to 0°C. Black arrows indicate where the A_R absorbance is at a maximum at low ΔT values, signifying end-fraying. This does not occur in the G_R kinetic traces as the GC bases primarily undergo melting rather than end-fraying in the **n-ODN** sequences.

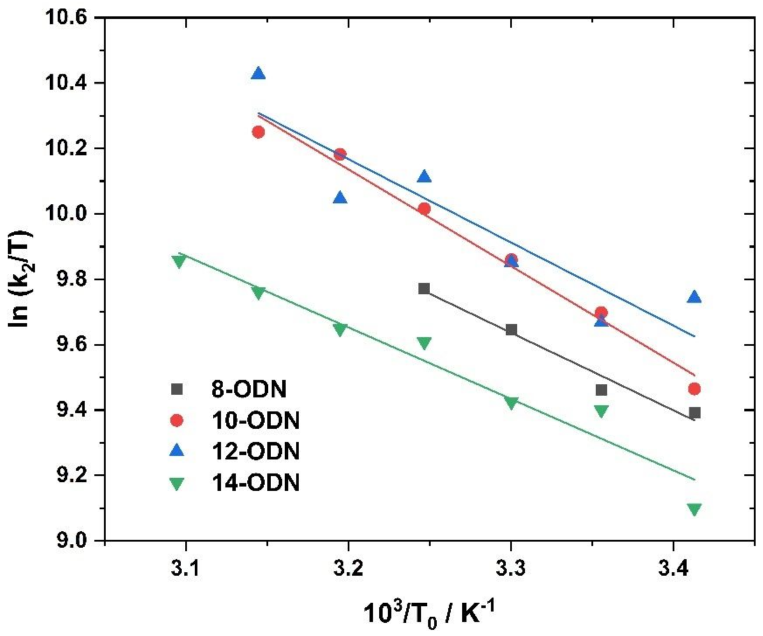

Figure S13. Eyring analysis of the temperature-dependent timescale of end-fraying (τ_2) determined via the A_R mode of **n-ODN** as a function of T_0. The maximum T_0 value used for this analysis is $T_0 = T_m$- 15°C, as above this, the absorbance of the A_R band is dominated by increase in absorbance due to melting (τ_3).

Table S3. Enthalpy of activation of end-fraying for **n-ODN** sequences as calculated from an Eyring analysis of the end-fraying (τ_2) timescale (Figure S4).

	**$\Delta H^{\ddagger}$ / kJ mol^{-1}**			
**n**	**8**	**10**	**12**	**14**
**n-ODN**	19.7 ± 0.3	24.6 ± 0.2	21.2 ± 0.6	18.2 ± 0.2

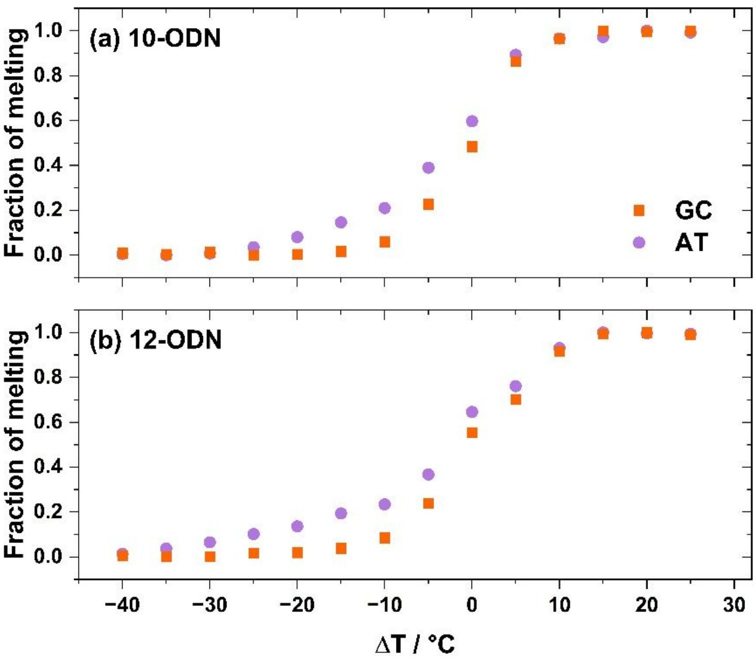

Figure S14. Melting curves derived from the change in intensity of the G_R (orange) and A_R (purple) modes for **10-ODN** (a) and **12-ODN** (b) as a function of ΔT. All datasets are normalized to their sigmoidal fit to show the fraction of melting.

Table S4. AT base pair end-fraying at ΔT = −20 °C as a function of **n-ODN**. Values were calculated by multiplying the fraction of melting of A_R by the number of AT base pairs in the **n-ODN** sequence.

	8-ODN	10-ODN	12-ODN	14-ODN
# AT base pairs frayed at ΔT = −20 °C	0.2	0.6	1.1	1.9

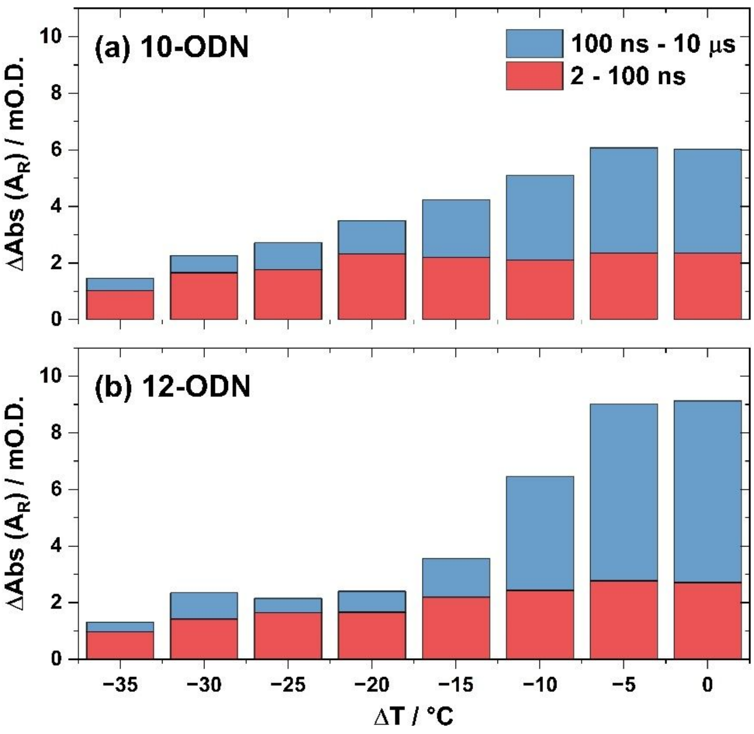

Figure S15. Comparison of rises in A_R absorbance as a function of ΔT for the T-jump IR experiments of **10-ODN** (b) and **12-ODN** (c) between 2 – 100 ns (red) and 100 ns–10 μs (blue).

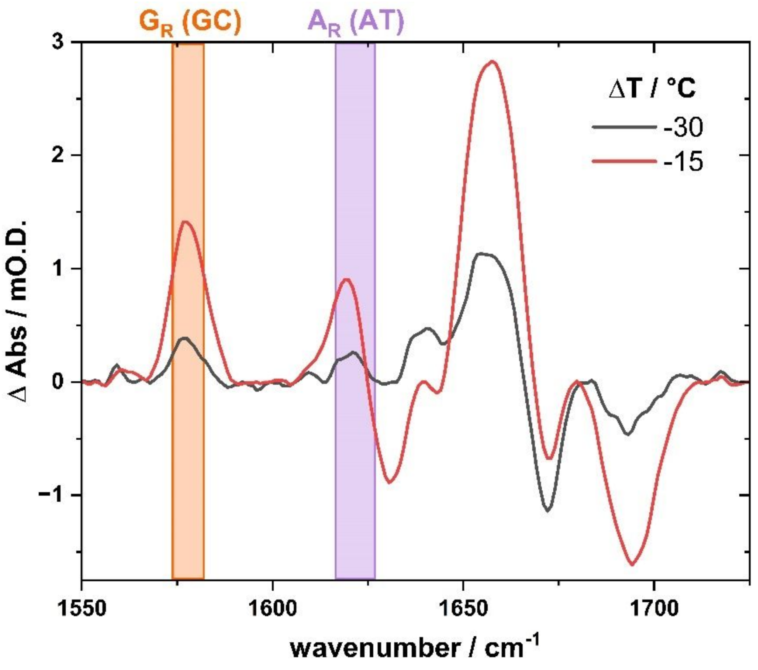

Figure S16. Difference FTIR absorption spectra for **PA1•8-ODN** following a 10°C increase with initial temperatures of ΔT = −30°C (black) and ΔT = −15°C (red). The G_R (orange) and A_R (purple) modes are highlighted.

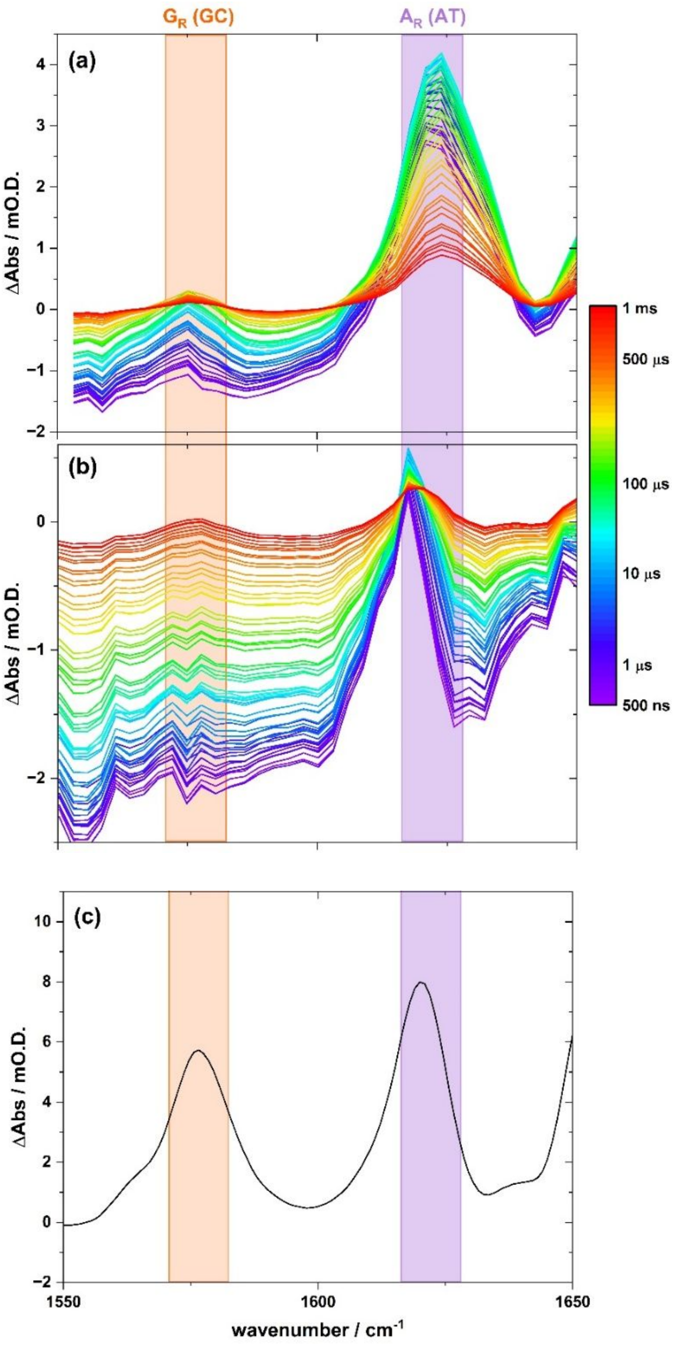

Figure S17. The growth of the G_R mode (indicative of melting) is shifted to later times in the T-jump IR spectra of **PA1•14-ODN** compared to **14-ODN**. T-jump IR spectra of **14-ODN** (a) and **PA1•14-ODN** (b) obtained as a function of T-jump-probe delay time from 500 ns to 1 ms at ΔT = −15°C. (c) Difference FTIR absorption spectra for **PA1•14-ODN** following a 10°C increase with initial temperatures of ΔT = −15°C, showing that melting does occur at this temperature in the complex. The G_R (orange) and A_R (purple) modes are highlighted.

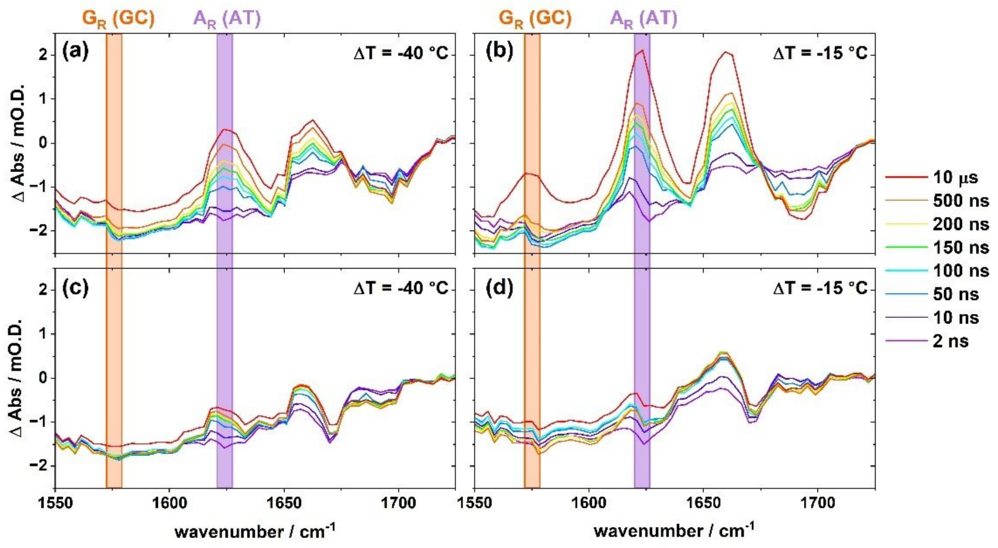

Figure S18. T-jump IR spectra of **10-ODN** (a and b) and **PA1•10-ODN** (c and d) obtained as a function of T-jump-probe delay time from 2 ns to 10 μs. Spectra were obtained at ΔT values of −40°C (a, c) and −15°C (b, d).

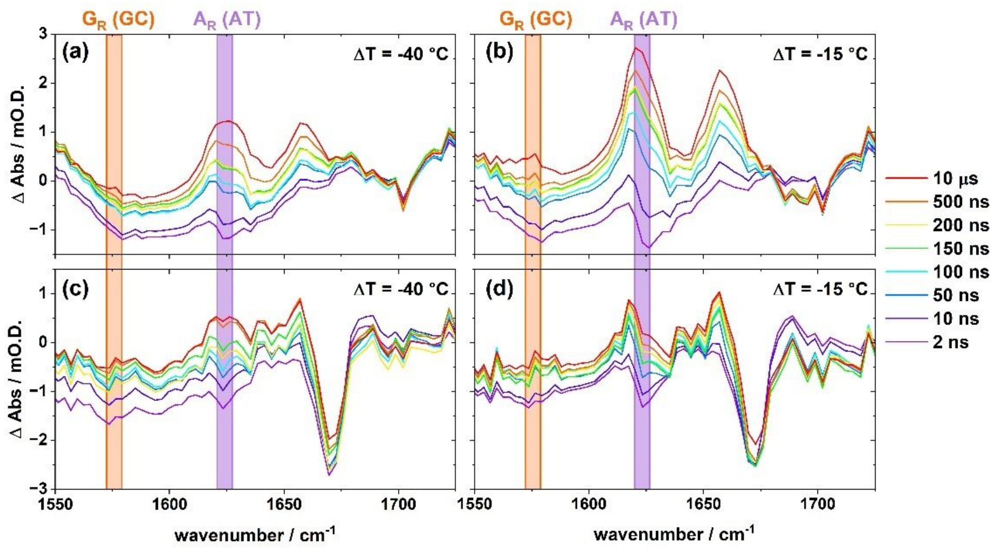

Figure S19. T-jump IR spectra of **12-ODN** (a and b) and **PA1•12-ODN** (c and d) obtained as a function of T-jump-probe delay time from 2 ns to 10 μs. Spectra were obtained at ΔT values of −40°C (a, c) and −15°C (b, d).

Table S5. Impact of **PA1** binding as a function of dsDNA length. Effect of sequence length and complex formation on the degree of dsDNA end-fraying at ΔT = -40°C. The degree of end-fraying is defined as $\frac{\Delta absorbance\ of\ A_R\ mode\ at\ 100\ ns\ (as\ measured\ using\ T\!-\!jump)}{\Delta absorbance\ of\ A_R\ mode\ under\ equilibrium\ conditions\ (as\ measured\ using\ FTIR)}$.

	**n-ODN**				**PA1•n-ODN**			
sequence length, n / b.p.	8	10	12	14	8	10	12	14
Degree of end-fraying	24	27	35	44	>1	5	8	13

Equation S1. The finite propagation length, ξ, of allosteric binding along DNA can be estimated assuming:

$$\Delta\mathrm{Abs}(x) = \Delta\mathrm{Abs}(0) \cdot \exp^{\frac{-|x|}{\xi}} \qquad (1)$$

where ΔAbs represents the decrease in the change in absorbance upon **PA1** binding and x indicates the number of base-pairs from the center of the binding site.

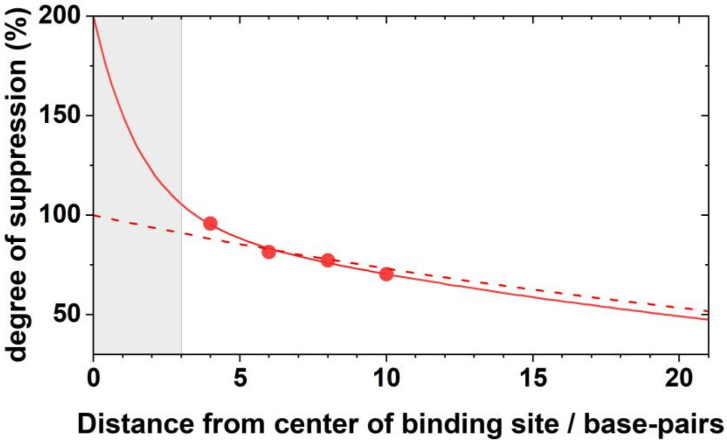

Figure S20. Suppression of end-fraying upon binding of PA1 to dsDNA (red points). Grey shaded area signifies the distance from the center of the PA1 binding site which still encompasses the **PA1** ligand. Fitting the data using Equation 1 gives an allosteric propagation length of 32 ± 4 base-pairs with an R^2 value of 0.8 (dotted red line). However, equation 1 assumes a point binding site, while **PA1** extends over 6 base pairs. Thus, fitting to a bi-exponential (solid red line) may be more suitable for this data set as point x=4 (**8-ODN**) is directly adjacent to the binding site. Using this alternative approach yields two decay parameters $\xi_1 = 1.6 \pm 0.1$ and $\xi_2 = 28 \pm 0.9$, where the latter is consistent with the long range allosteric impact obtained via Equation 1. Taking the average of the two long range decay parameters (28 and 32 bp) yields the figure of 30 bp quoted in the main text.

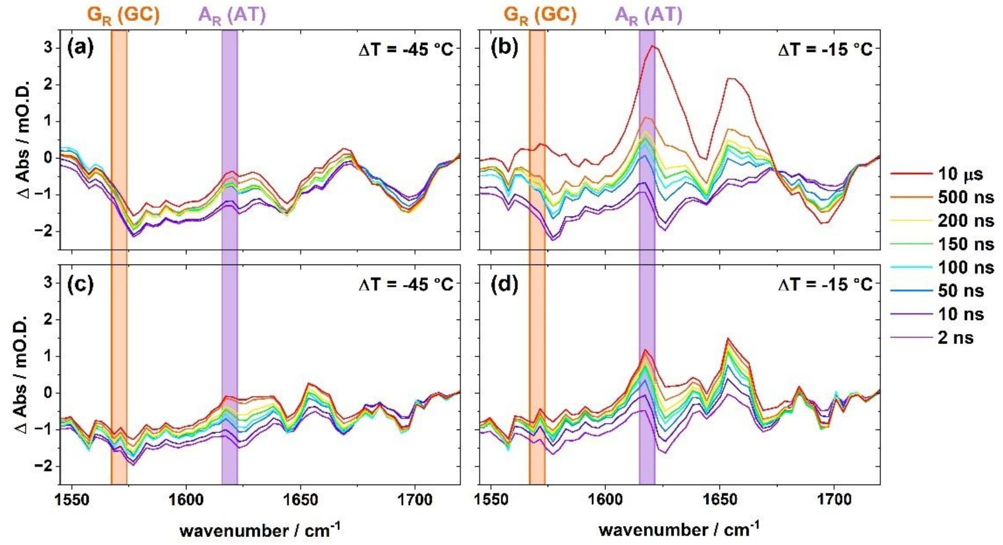

Figure S21. T-jump IR spectra of **14-ODN-left-fray** (a and b) and **PA1•14-ODN-left-fray** (c and d) obtained as a function of T-jump-probe delay time from 2 ns to 10 µs. Spectra were obtained at ΔT values of −45°C (a, c) and −15°C (b, d). **14-ODN-left fray** sequence is 5′-TATTAAATGTACAC-3′.

References

1. Padroni, G.; Parkinson, J. A.; Fox, K. R.; Burley, G. A. Structural basis of DNA duplex distortion induced by thiazole-containing hairpin polyamides. *Nucleic Acids Res.*, **2018**, *46*, 42-53.

2. Padroni, G.; Withers, J. M.; Taladriz-Sender, A.; Reichenbach, L. F.; Parkinson, J. A.; Burley, G. A. Sequence-Selective Minor Groove Recognition of a DNA Duplex Containing Synthetic Genetic Components. *J. Am. Chem. Soc.* **2019**, *141*, 9555–9563.

3. Greetham, G. M.; Clark, I. P.; Young, B.; Fritsch, R.; Minnes, L.; Hunt, N. T.; Towrie, M. Time-Resolved Temperature-Jump Infrared Spectroscopy at a High Repetition Rate. *Appl. Spectrosc.* **2020**, *74*, 720–727.
